# Health Care Costs After Genome-Wide Sequencing for Children With Rare Diseases in England and Canada

**DOI:** 10.1001/jamanetworkopen.2024.20842

**Published:** 2024-07-10

**Authors:** Deirdre Weymann, John Buckell, Patrick Fahr, Rosalie Loewen, Morgan Ehman, Samantha Pollard, Jan M. Friedman, Sylvia Stockler-Ipsiroglu, Alison M. Elliott, Sarah Wordsworth, James Buchanan, Dean A. Regier

**Affiliations:** 1 Cancer Control Research, BC Cancer Research Institute, Vancouver, British Columbia, Canada; 2 Faculty of Health Sciences, Simon Fraser University, Burnaby, British Columbia, Canada; 3 Health Economics Research Centre, Nuffield Department of Population Health, University of Oxford, Oxford, United Kingdom; 4 Nuffield Department of Primary Health Care Sciences, University of Oxford, Oxford, United Kingdom; 5 National Institute for Health Research Biomedical Research Centre, Oxford, United Kingdom; 6 Department of Medical Genetics, University of British Columbia, Vancouver, British Columbia, Canada; 7 BC Children’s Hospital Research Institute, Vancouver, British Columbia, Canada; 8 Department of Pediatrics, Faculty of Medicine, University of British Columbia, Vancouver, British Columbia, Canada; 9 Division of Biochemical Genetics, BC Children’s Hospital, Vancouver, British Columbia, Canada; 10 School of Population and Public Health, Faculty of Medicine, University of British Columbia, Vancouver, British Columbia, Canada

## Abstract

**Importance:**

Etiologic diagnoses for rare diseases can involve a diagnostic odyssey, with repeated health care interactions and inconclusive diagnostics. Prior studies reported cost savings associated with genome-wide sequencing (GWS) compared with cytogenetic or molecular testing through rapid genetic diagnosis, but there is limited evidence on whether diagnosis from GWS is associated with reduced health care costs.

**Objective:**

To measure changes in health care costs after diagnosis from GWS for Canadian and English children with suspected rare diseases.

**Design, Setting, and Participants:**

This cohort study was a quasiexperimental retrospective analysis across 3 distinct English and Canadian cohorts, completed in 2023. Mixed-effects generalized linear regression was used to estimate associations between GWS and costs in the 2 years before and after GWS. Difference-in-differences regression was used to estimate associations of genetic diagnosis and costs. Costs are in 2019 US dollars. GWS was conducted in a research setting (Genomics England 100 000 Genomes Project [100KGP] and Clinical Assessment of the Utility of Sequencing and Evaluation as a Service [CAUSES] Research Clinic) or clinical outpatient setting (publicly reimbursed GWS in British Columbia [BC], Canada). Participants were children with developmental disorders, seizure disorders, or both undergoing GWS between 2014 and 2019. Data were analyzed from April 2021 to September 2023.

**Exposures:**

GWS and genetic diagnosis.

**Main Outcomes and Measures:**

Annual health care costs and diagnostic costs per child.

**Results:**

Study cohorts included 7775 patients in 100KGP, among whom 788 children had epilepsy (mean [SD] age at GWS, 11.6 [11.1] years; 400 female [50.8%]) and 6987 children had an intellectual disability (mean [SD] age at GWS, 8.2 [8.4] years; 2750 female [39.4%]); 77 patients in CAUSES (mean [SD] age at GWS, 8.5 [4.4] years; 33 female [42.9%]); and 118 publicly reimbursed GWS recipients from BC (mean [SD] age at GWS, 5.5 [5.2] years; 58 female [49.2%]). GWS diagnostic yield was 143 children (18.1%) for those with epilepsy and 1323 children (18.9%) for those with an intellectual disability in 100KGP, 47 children (39.8%) in the BC publicly reimbursed setting, and 42 children (54.5%) in CAUSES. Mean annual per-patient spending over the study period was $5283 (95% CI, $5121-$5427) for epilepsy and $3373 (95% CI, $3322-$3424) for intellectual disability in the 100KGP, $724 (95% CI, $563-$886) in CAUSES, and $1573 (95% CI, $1372-$1773) in the BC reimbursed setting. Receiving a genetic diagnosis from GWS was not associated with changed costs in any cohort.

**Conclusions and Relevance:**

In this study, receiving a genetic diagnosis was not associated with cost savings. This finding suggests that patient benefit and cost-effectiveness should instead drive GWS implementation.

## Key Points

**Question:**

Is diagnosis from genome-wide sequencing associated with reduced health care costs for children with suspected rare diseases?

**Findings:**

In this cohort study of 3 groups of children who underwent genome-wide sequencing in England (7775 children in a research study) or Canada (118 children who received publicly funded sequencing and 77 children in a research study), diagnosis from genome-wide sequencing was not associated with changes in health care or diagnostic costs.

**Meaning:**

These findings suggest that sustainable clinical implementation of genome-wide sequencing must be motivated by evidence of patient and family benefit and cost-effectiveness rather than promises of cost savings from earlier diagnosis.

## Teaser

This cohort study investigates the association of diagnosis of rare diseases using genome-wide sequencing with health care casts among children in England and Canada.

## Introduction

As of 2020, there were more than 10 000 known rare diseases, which together affected 1 in 16 people worldwide.^1,2,3^ Rare diseases disproportionately affect children, with most such diseases caused by genetic factors. Identifying these underlying genetic causes, termed etiologic diagnoses, can inform prognosis and clinical management.^4,5^ With more than 7000 known gene-disease associations to consider and inefficiencies across testing pipelines, the mean time of search for a genetic etiology using genetic, cytogenetic, and genomic testing is currently between 4.8 and 7.4 years, costing health care systems more than $5000 per patient in laboratory tests alone.^2,6,7,8,9^ Time spent searching for an etiologic diagnosis, called the diagnostic odyssey, comprises repeated health care system interactions, inconclusive or null test results, misdiagnoses, and ineffective medical interventions.^1,10,11^ More than half of patients with rare diseases never receive an etiologic diagnosis even after accessing the most comprehensive testing available.^7,9^

Diagnostic testing for rare diseases can be done by a variety of methods, including karyotyping, fluorescence in situ hybridization, chromosomal microarray analysis, single-gene tests, and multigene (gene panel) tests.^12^ Genome-wide sequencing (GWS) can shorten the diagnostic odyssey for rare diseases, with improved diagnostic yield compared with sequential testing for specific subsets of genetic disorders.^4,13,14,15^ GWS includes sequencing all protein-coding regions of genes (whole-exome sequencing) or entire genomes (whole-genome sequencing).^16^ Published studies suggest that up-front GWS at symptom presentation is cost saving in patients with pediatric-onset neurodevelopmental disorders or in children with progressive neurological disorders through avoiding ineffective tests and treatments for patients who are diagnosed, based on retrospective data spanning patients’ entire diagnostic trajectories prior to GWS.^17,18^ While costs of GWS have decreased over time, the limited available evidence characterizing health care system outcomes is delaying GWS translation into clinical practice globally.^19,20^ The association of diagnosis with health care system expenditures is unknown. Providing evidence on these diagnostic outcomes in addition to outcomes associated with GWS is critical for informing resource allocation decisions throughout care continuums for patients and families living with rare diseases.

Our study examines costs before and after GWS in research and clinical settings for children with suspected rare diseases. We estimated associations of GWS diagnosis with health care expenditures. We drew on data from 2 countries where GWS is accessible to patients with suspected rare diseases: England and Canada. We focused on 2 clinical areas: developmental delay and seizure disorders. These common phenotypes frequently co-occur in patients with rare diseases and make up nearly half of all known rare disease–gene associations.^21,22,23,24,25^ Etiologic diagnosis for these conditions most often yields accurate information on prognosis, expected clinical course, and symptom management, such as through antiseizure medication, but can also allow for initiation of surveillance strategies or treatments targeting the underlying disease.^26,27,28,29^ Accurate diagnosis also supports genetic counseling for immediate family and at-risk extended family members. Pre- and post-test genetic counseling is recommended for all families undergoing GWS.^30^

In England, GWS was accessible to patients with suspected rare genetic disorders from 2014 to 2018 through the 100 000 Genomes Project (100KGP), a large-scale national sequencing program.^31,32^ In British Columbia (BC), Canada, the public health care system reimbursed GWS for diagnosing suspected genetic disorders in 2016 while evidence continued to emerge from ongoing research studies, and GWS remains clinically accessible.^33,34^ By examining multicountry economic outcomes across research and clinical applications, our results may inform translation of GWS from research to clinical practice.

## Methods

### Study Design

The University of British Columbia-BC Cancer Research Ethics Board and Genomics England approved and granted a waiver of consent for this cohort study, which involved the secondary use of previously collected, deidentified data. Reporting adheres to Strengthening the Reporting of Observational Studies in Epidemiology (STROBE) reporting guidelines. See eFigure 1 in Supplement 1 for our 2-country, retrospective cohort study design for analyzing historical genomic cohort data. In England, health care professionals from 9 English hospitals referred patients with suspected intellectual disability (ID), early-onset epilepsy (EoE), or both conditions to the 100KGP between 2014 and 2016.^32^ Patients underwent singleton-, duo-, trio-, or quad-based research whole-genome sequencing. Eligibility criteria specified a suspected rare disease with a likely single-gene or oligogenic cause and no current genetic diagnosis. Prior diagnostic testing was common but not required. In BC, our study included pediatric patients with developmental delay (DD), ID, seizures, or a combination of these conditions who (1) underwent trio-based GWS involving whole-genome sequencing or whole-exome sequencing between 2015 and 2018 through BC’s Clinical Assessment of the Utility of Sequencing and Evaluation as a Service (CAUSES) Research Clinic^33^ or (2) had a referral to the BC Children’s Hospital Division of Biochemical Genetics outpatient clinic in Vancouver, Canada, and received publicly reimbursed clinical GWS using whole-exome sequencing between 2016 and 2019. CAUSES received referrals from physicians across the province and required a strong suspicion of a single-gene disorder and the availability of both biological parents for trio testing.^33^ CAUSES additionally required at least 1 of the following: no genetic diagnosis from previous genetic, first-tier biochemical testing, or both (of a 2-tier process implemented as standard care in BC)^35^; a condition exhibiting genetic heterogeneity; or a family history suggestive of a Mendelian single-gene disorder. In contrast, patients who had a suspected genetic disorder were eligible for reimbursed clinical GWS if they completed prior standard care consultations and in-province tests and if testing offered clear potential for benefit. Requests included singleton, duo, or trio testing. We focused on ID and EoE in England and DD, ID, or seizures in BC based on data availability and disorder frequency. We consulted with 100KGP and BC Children’s Hospital clinicians to identify these patients in available records. Further cohort details are in the eMethods in Supplement 1.

We measured patient-level health care resource use and costs in England and diagnostic service use and costs in BC. Follow-up spanned 2 years before and 2 years after GWS, which we determined using data from the first interaction with the public health care system (excluding normal birth-related interaction) until death or the end of the study period (December 2019 in England and July 2019 in BC). The primary study end point was change in annual patient-level costs between pre-GWS and post-GWS periods. Periods were defined based on the date physicians returned GWS results to patients. We also estimated differential outcomes across patients who did or did not receive a genetic diagnosis from GWS. We converted costs to 2019 US dollars using country-specific inflation and exchange rates (1.2768 for Great British pounds and 0.7536 for Canadian dollars).

### Data Sources

In England, we identified eligible patients from the 100KGP dataset, which also included health care resource use data for secondary care (inpatient, outpatient, and emergency care) in the form of Hospital Episode Statistics data. We linked data to unit costs using National Health Service (NHS) costing data. Extracted data included patient characteristics, comorbidities, and diagnostic yield from sequencing based on the previously described 100KGP research-based variant classification pipeline.^32^ For British patients, race and ethnicity were derived from the 100KGP database, which captured information on the following race and ethnicity categories: Asian or Asian British (Bangladeshi, Indian, Pakistani, or any other Asian background), Black or Black British (African, Caribbean, or any other Black background), Chinese, White (British, Irish, or any other White background), multiracial or multiethnic (“mixed” in the database, consisting of White and Asian, White and Black African, White and Black Caribbean, or any other multiracial or multiethnic background), and any other racial or ethnic group. These are consistent with NHS classifications used in the UK. Patient race and ethnicity information is not routinely collected by Canada’s health care systems and could therefore not be abstracted from electronic health records for BC patients. Where data were available, we assessed participant race and ethnicity to inform equity in access to and outcomes associated with GWS. In BC, we identified eligible patients from CAUSES study documentation and departmental records. We manually abstracted information on patient characteristics, diagnostic yield from sequencing, which was determined using CAUSES variant classification pipelines and clinician notes,^36,37^ and annual health care resource use for provincially reimbursed diagnostic services (genetic, laboratory tests, imaging and physiological tests) from institutional and provincewide electronic health record systems (Cerner, CareConnect). We based diagnostic yield on variants that were pathogenic, likely pathogenic, or of uncertain significance and noted by clinicians as having contributed to patient phenotypes.^37^ We linked health care resource use data to unit costs from departmental and published sources. See the eMethods in Supplement 1 for additional data and costing details.

### Statistical Analysis

We conducted Canadian and English analyses in parallel after data acquisition in 2021. Data analysis was conducted from April 2021 to September 2023. Descriptive statistics characterized each cohort. Logistic regression assessed baseline differences in patient probability of diagnosis from GWS according to their observed characteristics, including age, sex, timing of GWS, phenotype, number of comorbidities, geographic location, race and ethnicity, and deprivation decile, depending on country data availability and including missing categories. We estimated annual per-patient costs across a range of upstream and downstream health care service categories. In England, categories included inpatient, outpatient, and emergency care. In Canada, where only diagnostic testing costs were observed, categories included genetic, imaging, physiological, and laboratory testing. We separated English analyses by condition (ID vs EoE). We pooled Canadian analyses across conditions because of small sample sizes but separated them by GWS setting (research vs publicly reimbursed). All cost analyses accounted for censoring arising from incomplete follow-up data using inverse probability of censoring weighting.^38,39^ We Winsorized cost outliers at the 99th percentile.

Pre-post analyses compared expenditures before and after GWS.^40^ We accounted for nonnormally distributed costs and repeated observations across patients using weighted mixed-effects generalized linear regression, specifying log-link and γ distributed outcomes. To estimate associations of patient characteristics with diagnosis, linear models fit difference-in-differences specifications. Difference-in-differences adjusts for unobserved baseline differences across diagnosed and undiagnosed groups, as well as unobserved factors that would be associated with parallel changes in costs in both groups over time.^41^ We tested the assumption of parallel outcomes trends through inclusion of interaction terms when analyzing costs before GWS. Both pre-post and difference-in-differences analyses measure the change in mean annual costs in the 2 years after GWS compared with the 2 years before GWS. Available sample sizes determined follow-up period length for base case analysis, with sensitivity to length examined in supplementary analyses.

Models adjusted for patient characteristics, calendar year fixed effects, continuous outcome trends, changes in mean costs after GWS, diagnosis group fixed effects, and individual random effects. Fixed effects capture observed and unobserved factors associated with costs that are constant within calendar year or within diagnosis group. Individual random effects incorporate patient-level variability in mean costs. Final models, reported in eTables 1 and 2 in Supplement 1, maximized goodness of fit according to Akaike and bayesian information criteria.^42^ In England, final pre-post and difference-in-differences models adjusted for age (continuously specified using natural splines), length of diagnostic odyssey (linearly specified), and categorical covariates of sex, race and ethnicity, census-based deprivation decile, and geographic region (based on Office for National Statistics classifications). In Canada, final models adjusted for outcome trends (linearly specified), year and group fixed effects, phenotype, number of comorbidities, age at GWS (continuously specified with a squared term), sex, urban vs rural geographic location, year of diagnostic odyssey in which GWS was accessed, and random effects. We conducted all analyses in Stata statistical software version 15 (StataCorp) or R statistical software version 4.0.2 (R Project for Statistical Computing).^43,44^ A threshold of *P* < .05 determined statistical significance.

## Results

### Descriptive Statistics

Study cohort sizes varied across jurisdictions. In England, there were 7775 participants in the 100KGP, including 788 children (10.1%) with EoE (mean [SD] age at GWS, 11.6 [11.1] years; 400 female [50.8%]; 79 Asian [10.0%], 19 Black [2.4%], and 520 White [66.0%]) and 6987 children (90.0%) with an ID (mean [SD] age at GWS, 8.2 [8.4] years; 2750 female [39.4%]; 656 Asian [9.4%], 135 Black [1.9%], and 4711 White [67.4%]), compared with 77 BC CAUSES Research Clinic participants (mean [SD] age at GWS, 8.5 [4.4] years; 33 female [42.9%]) and 118 recipients of BC’s publicly reimbursed GWS (mean [SD] age at GWS, 5.5 [5.2] years; median [IQR] age at GWS, 4 [2-9] years; 58 female [49.2%]). Adjusted for censoring, we observed patients over 64 088 person-years. Table 1 summarizes baseline characteristics for included patients. Phenotypes varied across cohorts. Among BC CAUSES patients, 51 individuals (66.2%) had a DD or an ID, 24 individuals (31.2%) had a DD or an ID and a seizure disorder, and 2 individuals (2.6%) had a seizure disorder. Among reimbursed GWS recipients, these proportions were 68 individuals (57.6%), 31 individuals (26.3%), and 19 individuals (16.1%), respectively.

**Table 1.** Study Cohort Characteristics

Baseline characteristic	Participants, No. (%)^a^			
	English 100KGP (N = 7775)		BC CAUSES Research Clinic (N = 77)	BC publicly reimbursed GWS (N = 118)
	EoE (n = 788)	ID (n= 6987)		
Proband status^b^				
Yes	695 (88.2)	6180 (88.4)	77 (100)	118 (100)
No	93 (11.8)	807 (11.6)	NA	NA
Sex				
Female	400 (50.8)	2750 (39.4)	33 (42.9)	58 (49.2)
Male	388 (49.2)	4237 (60.6)	44 (57.1)	60 (50.8)
Diagnostic times, y				
Age at earliest diagnostic service	mean, 0.2 (SD, 0.4)	mean, 0.7 (SD, 2.5)	median, 1 (IQR, 0-3)	median, 1 (IQR, 0-3)
Age at return of GWS results	mean, 11.6 (SD, 11.1)	mean, 8.2 (SD, 8.4)	mean, 8.6 (SD, 4.4)	median, 4 (IQR, 2-9)
GWS in proband diagnostic odyssey	mean, 11.4 (SD, 11.1)	mean, 7.5 (SD, 8.5)	median, 6 (IQR, 4-9)	median, 2 (IQR, 1-4)
Phenotype				
Developmental disorder, no seizure	NA	6987 (100)	51 (66.2)	68 (57.6)
Developmental and seizure disorder	NA	NA	24 (31.2)	31 (26.3)
Seizure disorder, no developmental disorder	788 (100)	NA	2 (2.6)	19 (16.1)
No. of concomitant disorders, mean (SD)	NA	NA	5.03 (2.15)	4.16 (1.76)
Race and ethnicity (self-reported)^c^				
Asian or Asian British	79 (10.0)	656 (9.4)	NA	NA
Black	19 (2.4)	135 (1.9)	NA	NA
Chinese	1 (0.1)	10 (0.1)	NA	NA
White	520 (66.0)	4711 (67.4)	NA	NA
Not known	128 (16.2)	1141 (16.3)	NA	NA
Multiracial or multiethnic	38 (4.8)	257 (3.7)	NA	NA
Other	9 (1.1)	77 (1.1)	NA	NA
Area of residence				
Urban	NA	NA	65 (84.4)	112 (94.9)
Rural	NA	NA	12 (15.6)	5 (4.2)
Missing	NA	NA	0	1 (0.8)
Deaths	0	1 (<0.1)	0	2 (1.7)
No. of samples sequenced				
Trio	NA	NA	77 (100)	20 (16.9)
Duo	NA	NA	0	5 (4.2)
Singleton	NA	NA	0	14 (11.9)
Missing	NA	NA	0	79 (66.9)
Diagnostic yield^d^	143 (18.1)	1323 (18.9)	42 (54.5)	47 (39.8)

Abbreviations: 100KGP, 100 000 Genomes Project; BC, British Columbia; BC CAUSES, British Columbia Clinical Assessment of the Utility of Sequencing and Evaluation as a Service; EoE, early-onset epilepsy; GWS, genome-wide sequencing; ID, intellectual disability; NA, not applicable.

^a^ Frequencies and percentages are reported for categorical variables, means and SDs for normally distributed continuous variables, and medians and IQRs for continuous variables showing evidence of nonnormality.

^b^ A proband is defined as an individual affected by a genetic disorder who is the first in their family to be affected.

^c^ Participants self-reported race and ethnicity according to the following subcategories: Asian or Asian British (Bangladeshi, Indian, Pakistani, or any other Asian background), Black (African, Caribbean, or any other Black background), Chinese, White (British, Irish, or any other White background), multiracial or multiethnic (“mixed” in the database, consisting of White and Black African, White and Black Caribbean, or any other multiethnic or multiracial background), other (any other ethnic group), and not known.

^d^ Diagnostic yield was based on variants that were pathogenic, likely pathogenic, or of uncertain significance and noted by clinicians as having contributed to patient phenotypes.^37^

Timing of GWS and diagnostic outcomes also differed. In England, patients with EoE accessed GWS at a mean (SD) of 11.4 (11.1) years into their diagnostic odyssey compared with 7.5 (8.5) years for patients with ID. In a BC research setting, patients strongly suspected of having a single-gene disorder accessed GWS at a mean (SD) of 6.8 (3.9) years (median [IQR], 6 [4-9] years) into their odyssey. In the BC publicly reimbursed clinical setting, access occurred earliest, a mean (SD) of 3.1 (3.0) years (median [IQR], 2 [1-4] years) into the patient odyssey. In the 100KGP, the diagnostic yield of GWS was 143 children (18.1%) for EoE and 1323 children (18.9%) for ID. Diagnostic yield was highest among BC CAUSES Clinic research participants, estimated at 42 children (54.5%). In the BC reimbursed setting, 47 children (39.8%) received a diagnosis. Logistic regressions revealed few significant baseline differences across patients who were diagnosed and not diagnosed in any cohort (eTables 3 and 4 in Supplement 1).

### Health Care Spending

**Figure 1.**
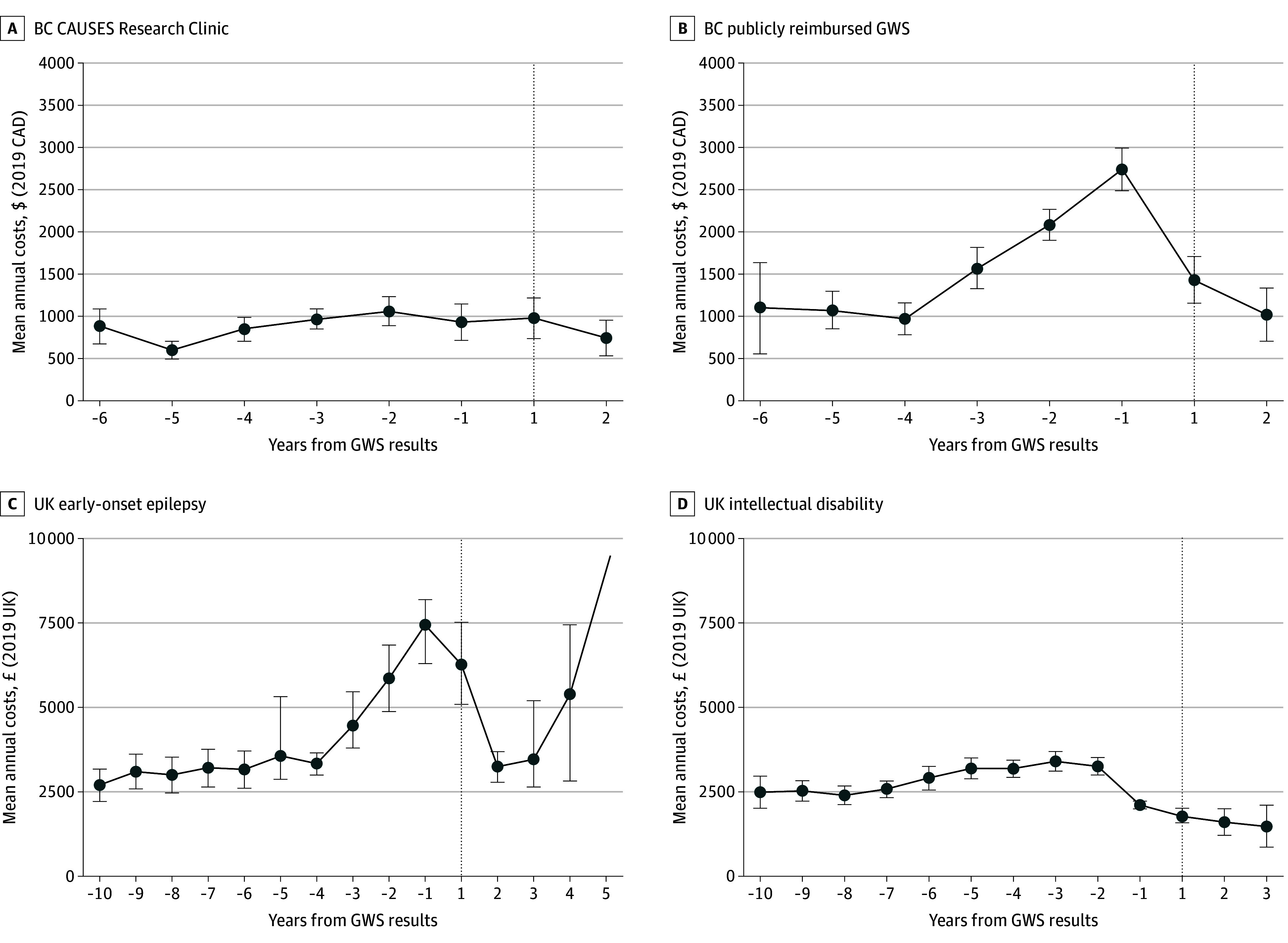
Unadjusted Annual Total Cost Trajectory Across Cohorts Mean annual costs unadjusted for covariates or censoring are reported in 2019 Canadian dollars or UK pounds sterling for complete cases only. BC indicates British Columbia; BC CAUSES, British Columbia Clinical Assessment of the Utility of Sequencing and Evaluation as a Service; GWS, genome-wide sequencing.

Patient unadjusted cost trajectories are in Figure 1 and eFigure 2 in Supplement 1, with unadjusted means reported in eTables 5 and 6 in Supplement 1. In England, the mean total annual per-patient spend over the 4-year period (2 years pre-GWS testing and 2 years post-GWS testing) was higher for EoE than ID, at $5283 (95% CI, $5121-$5427) vs $3373 (95% CI, $3322-$3424). In BC, the mean total annual per-patient costs for diagnostic services were lower for research-based GWS compared with publicly reimbursed GWS recipients, at $724 (95% CI, $563-$886) vs $1573 (95% CI, $1372-$1773). These trends held across health service categories (inpatient, outpatient, and emergency care in England and genetic, imaging, and physiologic, and laboratory testing in BC).

### Changes in Health Care Spending After GWS

Adjusting for censoring and confounding, pre-post analyses (Table 2 and Table 3) showed increases in mean total annual per-patient spend in England for EoE ($1185; 95% CI, $11 to $2358; *P* = .047) and ID ($273; 95% CI: $161 to $386, *P* < .001) after GWS compared with before testing. This difference varied across health service categories. For EoE, the pre-post–GWS difference in mean annual per-patient spend was not significant for inpatient care ($282; 95% CI, −$716 to $1281; *P* = .59) or emergency care ($54; 95% CI, −$5 to $112, *P* = .08) but showed an increase of $641 (95% CI, $294 to $988; *P* < .001) for outpatient care. For ID, the pre-post–GWS difference in mean annual per-patient spend was $112 (95% CI, $63 to $162; *P* < .001) for inpatient care, $200 (95% CI, $154 to $246; *P* < .001) for outpatient care, and $18 (95% CI, $13 to $23; *P* < .001) for emergency care. In BC, where we observed only diagnostic testing costs, there was no statistically significant difference in total annual diagnostic costs after research-based GWS ($405; 95% CI, −$185 to $996; *P* = .18) vs before GWS. In contrast, annual per-patient diagnostic costs declined by $1048 (95% CI, −$1722 to −$375; *P* = .002) after publicly reimbursed GWS vs before GWS. Significant reductions in genetic and laboratory testing were the major contributors to diagnostic cost reductions after publicly reimbursed GWS. Examining alternate pre- and post-GWS period lengths showed similar results (eTables 7 and 8 in Supplement 1).

**Table 2.** Pre-Post and Difference-in-Differences Estimates of Change in Costs, Canada

Outcome model	BC publicly reimbursed GWS (N = 118; diagnostic yield = 24 [39.8%])		BC CAUSES Research Clinic (N = 77; diagnostic yield = 42 [54.5%])	
	Cost estimate (SE), US $	*P* value	Cost estimate (SE), US $	*P* value
**Pre-post change after GWS** ^a^				
Annual diagnostic costs over 2 y before and 2 y after GWS	−1048 (344)	.002	405 (301)	.18
Genetic testing	−1027 (414)	.01	−47 (29)	.10
Imaging and physiological testing	−229 (157)	.14	288 (147)	.05
Laboratory testing	−473 (173)	.006	69 (96)	.47
**Difference-in-differences estimate of association of GWS diagnosis with annual costs** ^b^				
Annual diagnostic costs over 2 y before and 2 y after GWS	56 (348)	.87	−555 (295)	.06
Genetic testing	−147 (127)	.25	−100 (118)	.40
Imaging and physiological testing	99 (141)	.48	−146 (96)	.13
Laboratory testing	127 (188)	.51	−317 (154)	.04

Abbreviations: BC, British Columbia; BC CAUSES, British Columbia Clinical Assessment of the Utility of Sequencing and Evaluation as a Service; GWS, genome-wide sequencing.

^a^ SEs were estimated using the δ method. Estimates are adjusted for individual random effects, outcome trends, time fixed effects, and baseline covariates. The significance level was *P* < .05.

^b^ SEs are corrected for clustering at the individual level. Estimates are adjusted for time and group fixed effects and baseline covariates. The significance level was *P* < .05.

**Table 3.** Pre-Post and Difference-in-Differences Estimates of Change in Costs, England

Outcome model	English 100KGP			
	EoE (n = 788; diagnostic yield = 143 [18.1%])		ID (n = 6987, diagnostic yield = 1323 [18.9%])	
	Cost estimate (SE), US $	*P* value	Cost estimate (SE), US $	*P* value
**Pre-post change after GWS** ^a^				
All care over 2 y before and 2 y after GWS	1185 (599)	.05	273 (57)	<.001
Inpatient	282 (509)	.59	112 (26)	<.001
Outpatient	641 (177)	<.001	200 (23)	<.001
Emergency	54 (31)	.08	18 (3)	<.001
**Difference-in-differences estimate of association of GWS diagnosis with annual costs** ^b^				
All care over 2 y before and 2 y after GWS	444 (710)	.54	−110 (105)	.30
Inpatient	255 (568)	.67	−143 (96)	.14
Outpatient	192 (203)	.35	11 (41)	.79
Emergency (accident and emergency)	−9 (34)	.79	−5 (6)	.47

Abbreviations: 100KGP, 100 000 Genomes Project; EoE, early-onset epilepsy; GWS, genome-wide sequencing; ID, intellectual disability.

^a^ SEs were estimated using the δ method. Estimates are adjusted for individual random effects, outcome trends, time fixed effects, and baseline covariates. The significance level was *P* < .05.

^b^ SEs are corrected for clustering at the individual level. Estimates are adjusted for time and group fixed effects and baseline covariates. The significance level was *P* < .05.

### Differences in Health Care Spending From Diagnosis

**Figure 2.**
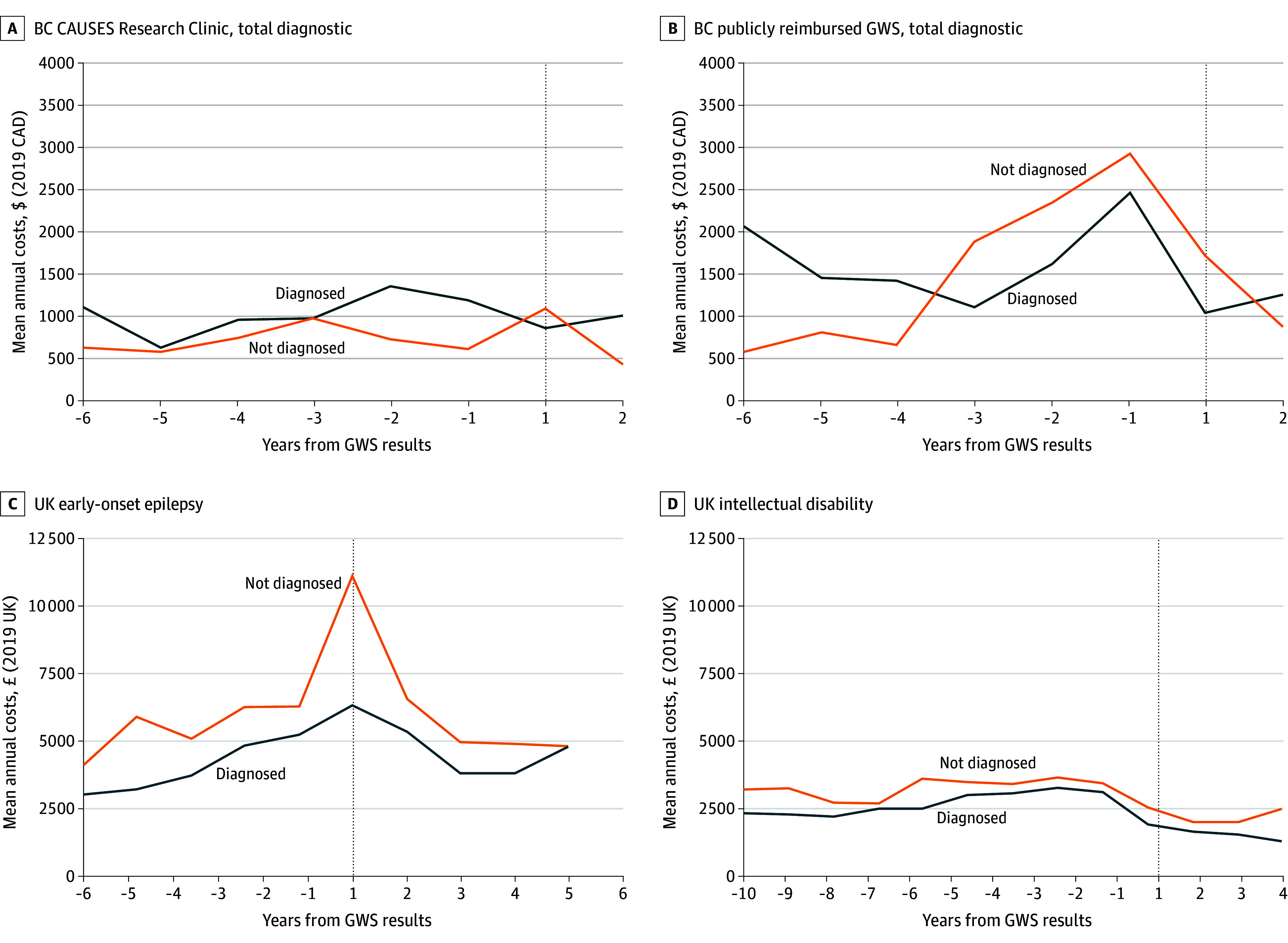
Unadjusted Annual Total Cost Trajectory Across Cohorts by Diagnosis Mean annual costs unadjusted for covariates or censoring by genome-wide sequencing (GWS) diagnosis are reported in 2019 Canadian dollars or UK pounds sterling for complete cases only. BC indicates British Columbia; BC CAUSES, British Columbia Clinical Assessment of the Utility of Sequencing and Evaluation as a Service; GWS, genome-wide sequencing.

Unadjusted cost trajectories stratified according to patient test results are shown in Figure 2 and eFigure 3 in Supplement 1. Difference-in-differences estimates for the 4-year period are reported in Table 2 and Table 3 and indicate the change in costs after GWS vs before GWS for patients with vs without a diagnosis. After adjusting for censoring and confounding, costs in the presequencing period suggested parallel trends in all cohorts. In England, pre-post–GWS changes in annual patient-level costs did not vary by diagnostic yield for EoE ($444; 95% CI, −$946 to $1834; *P* = .54) or ID (−$110; 95% CI, −$314 to $94; *P* = .30). In the BC research setting, pre-post–GWS costs for patients who were diagnosed vs undiagnosed were not statistically significantly different (−$555; 95% CI, −$1141 to $32; *P* = .06). Among cost categories, we observed significantly lower costs after GWS only in laboratory testing (−$317; 95% CI, −$623 to −$11; *P* = .04). In the publicly reimbursed setting, there were no significant differential outcomes in pre-post–GWS costs by genetic diagnosis ($56; 95% CI, −$634 to $746; *P* = .87). Shortening or extending the study period did not substantively change results (eTables 7 and 8 in Supplement 1).

## Discussion

In this cohort study, we estimated associations of GWS diagnosis with English health care costs and Canadian diagnostic testing costs for children with rare diseases, focusing on patients with developmental and seizure disorders. Rates of GWS diagnosis varied across cohorts depending on eligibility criteria, timing, and specific application of GWS. In the presence of varying diagnostic yields, we did not find evidence that receiving a genetic diagnosis was associated with reduced costs except for in a BC research setting. In that setting, highly selected patients accessed GWS without having to first exhaust available biochemical tests and could continue laboratory testing after a null GWS result.^33^ We found some evidence that undergoing GWS was associated with changes in health care costs irrespective of patient diagnosis. In a Canadian publicly reimbursed health care system where patients accessed GWS earlier in their diagnostic odyssey, we observed lower diagnostic testing costs after GWS, whereas no changes occurred in a Canadian research setting. In the English publicly reimbursed health care system, we observed increased inpatient and outpatient health care costs after GWS, with estimated magnitudes varying by clinical condition. These multicountry findings suggest that potential diagnostic cost savings from ending the search for a diagnosis after GWS and avoiding further, redundant testing may be offset by other increasing areas of health care use, although this phenomenon must be further verified in comparative research. GWS test results and rare disease diagnosis are unlikely to be associated with differential outcomes after implementation.

A key strength of our study was generating evidence across 2 countries, which gave evidence from 2 comparable health care systems and from different settings within those systems. Detailed information on diagnostic testing histories was available in Canada, capturing all genetic, laboratory, imaging, and physiological tests supported by the province, whereas complete administrative hospitalization data were available in England, enabling cost analysis across a range of care settings. Canadian data also captured patients receiving GWS only in research or clinically reimbursed GWS, allowing for estimation of GWS-associated outcomes across research and clinical settings, a comparison that reveals heterogeneous results and is missing from published literature.^45^ Our difference-in-differences analysis further built on past correlative cost evaluations^18,46^ by adjusting for unobserved baseline outcome differences and group-invariant time trends and when estimating associations between genetic diagnosis and health care costs.

To date, investigations into the clinical and economic value of genomics for diagnosing rare diseases primarily estimate incremental costs per additional diagnosis.^47,48,49^ While past research indicated that stakeholders value outcomes after diagnosis and that diagnostic yield from GWS can change when sequencing data are reanalyzed,^36,50,51,52^ downstream outcomes associated with GWS for patients and health care systems are uncertain. In infants who are critically ill, associations of GWS with use outcomes are mixed, with matched case study analysis showing cost reductions and propensity score–weighted analysis showing no differences compared with non-GWS testing.^53,54^ For patients with IDs, mean health care costs were 80% lower per patient after whole-exome sequencing than before sequencing irrespective of diagnostic outcome, but the authors did not establish statistical significance of these results.^55^ For children with structural malformations, unexplained DDs or IDs, or both, downstream health care service composition was different after whole-genome sequencing compared with chromosomal microarray, but there was no adjustment for costs preceding testing, potentially biasing results.^46^

Our study builds on this past research by explicitly controlling for baseline cost differences preceding GWS and assessing associations of GWS testing and resulting genetic diagnoses with health care costs. While clinical and regional heterogeneity will be associated with the magnitude of cost differences detected, combined evidence demonstrates that rare disease diagnosis is not associated with diagnostic cost savings.^17,18^ Instead, we observed changes in health care system costs after patients accessed GWS. Downstream changes may reflect uptake of more targeted medical interventions and other changes in clinical management previously documented after sequencing, from which patients may benefit.^22,56^ Evaluations that focus on intermediate diagnostic end points are unable to capture these downstream outcomes and so alone are insufficient to inform clinical implementation. Additional research is necessary to explore downstream patient and family benefits from changing health care use alongside cost outcomes associated with GWS.

### Limitations

Our findings must be interpreted in light of study limitations. In both jurisdictions, the assumed index date defining pre- and post-GWS periods was the date physicians returned test results to patients and their families. Mean turnaround times for GWS varied by cohort so costs accrued between the date of GWS blood draw and return of results will influence estimates. Our index date reflects when GWS results would be available to impact patient care. Applying an alternative index date may yield different results. We also restricted our follow-up period for comparison of downstream costs to at most 2 years after GWS based on available sample sizes, and it is possible that further changes in health care resource use occur over a longer time. Measuring longer-term costs and exploring outcomes across key dates in patient diagnostic trajectories is an important area for further research.

Difference-in-differences analysis requires that outcomes follow parallel trends across groups in the absence of diagnosis, an untestable assumption. To assess plausibility, we analyzed pre-GWS cost trends and failed to detect nonparallel trends in the presequencing period. These results provide support for the parallel trends assumption but do not guarantee it.^57^ We recognize other threats to the associations measured via pre-post and difference-in-differences analyses, such as selection bias of individuals participating in the English and Canadian studies. The pre-post analysis includes no counterfactual, and future comparative outcome assessment among GWS and non-GWS recipients is needed to understand outcomes associated with GWS. An additional limitation involves the lack of comparable cost data across jurisdictions that capture different types of health service use and are from health care systems with different pricing arrangements.^58,59^ In English Hospital Episode Statistics data, we costed individual episodes from routinely collected data that were not designed for research purposes and can be imprecise. While Hospital Episode Statistics data are the most accurate resource use data available, they do not contain the same detailed information on diagnostic costs as is available in the Canadian data. In Canada, cost data captured all in-province diagnostic service use for our study cohorts, but sample sizes were small, limiting our ability to stratify analyses by conditions and the statistical efficiency of point estimates. Both countries are high income, and all analyses focused on only developmental and seizure disorders. Future research examining generalizability to other rare disease conditions and jurisdictions, particularly low- and middle-income countries, is essential.

## Conclusions

GWS has important implications for patients, families, and health care systems. In this cohort study among English and Canadian children who underwent GWS for a suspected rare disease, we observed changing trends in post-GWS costs compared with pre-GWS costs. However, GWS test results and rare disease diagnosis were not associated with promised cost savings for health care systems. These findings suggest that cost minimization alone cannot justify translation of GWS into health care systems. Instead, evidence of patient and family benefit from diagnosis and cost-effectiveness based on all health care service use must guide global implementation decisions.

## Notes

Supplement 1.
eMethods.
**eFigure 1.** Study design diagram **eTable 1.** Difference-in-difference regression estimates for 2 years before and 2 years after in BC cohorts **eTable 2.** Difference-in-difference regression estimates for 2 years before and 2 years after in English cohorts **eTable 3.** Logistic regression of probability of diagnosis from GWS, Canadian cohorts **eTable 4.** Logistic regression of probability of diagnosis from GWS, UK cohorts **eFigure 2.** Unadjusted annual total cost trajectory across cohorts **eTable 5.** Unadjusted annual costs over 2 years before and 2 years after period, Canada **eTable 6.** Unadjusted annual costs over 2 years before and 2 years after period, England **eFigure 3.** Unadjusted annual total cost trajectory across cohorts, stratified according to diagnosis from GWS **eTable 7.** Sensitivity analysis considering 1 year before and 1 year after GWS **eTable 8.** Sensitivity analysis considering 6 years before and 2 years after GWS
eReferences.

Supplement 2.
Data Sharing Statement
