# Supplementary material for: Health Care Costs After Genome-Wide Sequencing for Children With Rare Diseases in England and Canada
Source: JAMA Netw Open. 2024 Jul 10;7(7):e2420842. doi: 10.1001/jamanetworkopen.2024.20842 (PMC11238031; doi:10.1001/jamanetworkopen.2024.20842)
Supplement: Supplement 1. — eMethods. eFigure 1. Study design diagram eTable 1. Difference-in-difference regression estimates for 2 years before and 2 years after in BC cohorts eTable 2. Difference-in-difference regression estimates for 2 years before and 2 years after in English cohorts eTable 3. Logistic regression of probability of diagnosis from GWS, Canadian cohorts eTable 4. Logistic regression of probability of diagnosis from GWS, UK cohorts eFigure 2. Unadjusted annual total cost trajectory across cohorts eTable 5. Unadjusted annual costs over 2 years before and 2 years after period, Canada eTable 6. Unadjusted annual costs over 2 years before and 2 years after period, England eFigure 3. Unadjusted annual total cost trajectory across cohorts, stratified according to diagnosis from GWS eTable 7. Sensitivity analysis considering 1 year before and 1 year after GWS eTable 8. Sensitivity analysis considering 6 years before and 2 years after GWS eReferences. [file jamanetwopen-e2420842-s001.pdf]

Supplemental Online Content

Weymann D, Buckell J, Fahr P, et al. Health care costs after genome-wide sequencing for children with rare diseases in England and Canada. *JAMA Netw Open*. 2024;7(7):e2420842. doi:10.1001/jamanetworkopen.2024.20842

eMethods.

eFigure 1. Study design diagram

eTable 1. Difference-in-difference regression estimates for 2 years before and 2 years after in BC cohorts

eTable 2. Difference-in-difference regression estimates for 2 years before and 2 years after in English cohorts

eTable 3. Logistic regression of probability of diagnosis from GWS, Canadian cohorts

eTable 4. Logistic regression of probability of diagnosis from GWS, UK cohorts

eFigure 2. Unadjusted annual total cost trajectory across cohorts

eTable 5. Unadjusted annual costs over 2 years before and 2 years after period, Canada

eTable 6. Unadjusted annual costs over 2 years before and 2 years after period, England

eFigure 3. Unadjusted annual total cost trajectory across cohorts, stratified according to diagnosis from GWS

eTable 7. Sensitivity analysis considering 1 year before and 1 year after GWS

eTable 8. Sensitivity analysis considering 6 years before and 2 years after GWS

eReferences.

This supplemental material has been provided by the authors to give readers additional information about their work.

© 2024 Weymann D et al. *JAMA Network Open.*

eMethods.

Study Criteria

100KGP Cohort

In England, patients were referred by their healthcare professionals to 100KGP and recruited from nine English hospitals.[1] Patients were eligible if they were suspected to have rare diseases with a likely single gene or oligogenic cause and had not yet received a genetic diagnosis. Patients often had a history of genetic and non-genetic investigations, including single gene tests, karyotyping, single-nucleotide polymorphism arrays, multi-gene panels, or exome sequencing. Where feasible, parents or other family members were enrolled in 100KGP to support duo or trio testing.

The 100KGP classified EoE patients into epilepsy categories and ID patients into ID categories based on a combination of assessment criteria and clinical expertise. EoE patients were identified as belonging to epilepsy categories in the Genomics England dataset and having an age of onset up to and including 48 months old.[2] ID patients were identified as belonging to the ID categories in the Genomics England dataset. Patients not classified as having EoE or ID were excluded from the analysis.

CAUSES Cohort

In British Columbia, Canada, patients were referred to CAUSES by their physicians. Referring physicians included general practitioners, pediatric specialists and subspecialists, and medical genetics physicians from across the province, although medical genetics, metabolic disease/biochemical genetics, and neurology were the most common referring disciplines.[3,4] Patients were selected for research-based GWS through CAUSES if they were strongly suspected to have a single gene disorder, had both biological parents available for trio testing, and met at least one of the following additional criteria: previous sequential genetic testing, including chromosomal microarrays, single-gene or multi-gene tests, and first tier biochemical testing did not identify a genetic cause; condition exhibited extensive genetic heterogeneity; and/or family history was suggestive of a Mendelian single-gene disorder. Anticipated low diagnostic yield was an exclusion criterion.

BC Publicly Reimbursed GWS Cohort

Reimbursement of GWS occurs on a case-by-case basis in BC and requires submission of an application justifying the need for testing and expected implications for patient care. Patients were eligible to apply for publicly reimbursed GWS if they had a suspected genetic disorder, had

© 2024 Weymann D et al. *JAMA Network Open.*

completed prior consultations and in-province tests, and testing offered potential for clear patient benefit (e.g., through treatment change).[5]

Canadian Data Sources

To assess cohort inclusion, information on genomic testing, patient demographics and phenotypic characteristics captured in departmental referrals and the BC Children's Hospital (BCCH) institutional EMR system (Cerner) was manually reviewed by four coders (JD, HC, ME, FM). Initially, patient records were reviewed in duplicate to assess concordance. Any coding discrepancies that emerged were resolved by the coders and when necessary, through consultation with the larger project team. Ineligible phenotypes were then excluded. Owing to the infeasibility of conducting extensive manual record reviews for a large cohort, we randomly selected 20% of CAUSES Clinic participants for consideration. Following initial review, we excluded 39 (34%) of CAUSES Clinic participants and 192 (62%) of clinical patients owing to ineligible phenotypes, location of residence (outside of BC), or denial of GWS requisition.

Coders accessed both the BCCH institutional EMR system and the BC province-wide EMR system (CareConnect) to comprehensively capture diagnostic services rendered during the study period. Data was gathered from first interaction with the public healthcare system (excluding normal birth-related interaction), until death or end of the study period (July 2019 in BC). In BC, data spanned up to 8 years prior and 3 years after GWS, although sample sizes were greatly reduced in the years furthest removed from GWS. Eligible diagnostic services included diagnostic imaging, physiological tests, specimen collection, genetic testing and laboratory testing. Coders abstracted GWS information pertaining to service dates, turnaround times, test type (e.g., whole exome or whole genome sequencing, singleton or trio) and GWS results (e.g., pathogenic variant, variant of uncertain significant (VUS), secondary findings) recorded by clinicians within patient EMRs. Patient characteristics, including sex, age, and geographic region of residence were determined based on clinician notes. Rural and urban locations were determined using Statistics Canada definitions for rural postal codes and small population centres.[6,7]

Canadian Costing

Costing was performed using the Canadian Agency for Drugs and Technologies in Health's (CADTH) guidelines for the costing of healthcare resources in a Canadian setting.[8] Unit costs of diagnostic services were identified based on: the Ministry of Health Medical Services Commission Payment Schedule[9]; the Ministry of Health Schedule of Fees for Laboratory Services[10]; the B.C. Ministry of Health MSP Fee-For-Service Payment Analysis 2016/2017 – 2020/2021;[11] departmental tracking from BCCH, literature sources and publicly listed information from the following laboratories in Canada: Lifelabs Genetics, Pacific Fertility, Initio

© 2024 Weymann D et al. *JAMA Network Open.*

Medical, Prevention Genetics, and Canada Diagnostic. When public, published, or commercial prices for laboratory tests within Canada were not readily available, U.S. prices were researched on www.findlabtest.com. U.S. prices were converted into Canadian Dollars using a 2019 Bank of Canada conversion rate of CAD$1.32 per USD$1.00. Unit costs and corresponding sources are provided in the eMethods Table. All unit costs were converted to 2019 Canadian Dollars using Bank of Canada CPI inflation rates.

© 2024 Weymann D et al. *JAMA Network Open.*

eMethods Table: Canadian unit costs for diagnostic services

Item	Cost	Cost Source
**IMAGING**		
Computed Radiography – CR	49.82	MSP
Computed Tomography – CT	98.44	MOH combined
Digital Radiography – DR	49.82	MSP
Nuclear Medicine –NM	262.66	MOH combined
PET/PT	2750.00	Commercial
Ultrasound –US	89.86	MOH combined
X-ray	36.90	MOH combined
Fluoroscopy – RF	40.58	MSP
MRI	863.39	Canada Diagnostic Fee 2020
MRI brain	863.39	Canada Diagnostic Fee 2020
Other Imaging	137.90	MOH combined
**PHYSIOLOGIC**		
Arthrogram (joint imaging)	41.80	MOH combined
Bone Mineral Densitometry	79.80	MOH combined
Brainstem and Auditory Evoked Potential Test (audiometry)	48.66	MOH combined
Echo cardiogram	187.35	MOH combined
Electrocardiogram	35.43	MOH combined
Electrocardiogram 24 hr	99.42	MOH combined
Electro-encephalogram (EEG)	127.80	MOH combined
Electro-encephalogram (EEG) 24 hr	2016.85	Dragojlovic, et al.[14]
Endoscopy	222.42	MOH combined
Nerve Conduction Study (NCS) or Electromyography (EMG)	101.71	MOH combined
Ophthalmic Evaluation	41.70	MOH combined
Spirometry (Respiratory Therapy)	15.84	MOH combined
Visual Evoked Potential Test	71.89	MOH
Polysomnography (standard)	554.42	MOH combined
Polysomnography (two-night)	1025.14	MOH combined
Multiple Sleep Latency Test	277.21	MOH combined
Other – Physiologic	309.21	Average of above
**SPECIMEN COLLECTION**		
Blood specimen	5.92	MOH
CSF Specimen	82.49	MOH combined
Nasopharyngeal or FLOQSwab specimen	18.20	MSP
Other Surgical Specimen (e.g. tonsil)	42.70	Average cost within category
Saliva Specimen	42.70	Average cost within category
Skin Specimen	51.66	MOH
Stool Specimen	16.92	MSP
Tracheal Aspirate Specimen	81.00	MOH
Urine Specimen	42.70	Average cost within category
Other specimen (average)	42.70	Average cost within category

© 2024 Weymann D et al. *JAMA Network Open.*

Item	Cost	Cost Source
**LAB TESTS**		
25-Hydroxyvitamin D (or 1-25)	94.35	MOHL
7-dehydrocholesterol	198.42	MOHL
Acetylcholine Receptor Ab	134.51	MOHL
Acetyl-CoA-glucosamine N-Acetyl transferase (MPS IIIc)	93.57	MOHL
ACTH (Adrenocorticotropic Hormone) Stimulation Test	45.17	MOHL
Acylcarnitine Panel or Profile	41.22	MOHL
Adenovirus, Rotavirus, Norovirus, Enterovirus	105.24	MOHL
Alanine Aminotransferase	1.47	MOHL
Albumin (all specimens apart from CSF)	1.57	MOHL
Albumin; CSF	20.13	MOHL
Albumin/Creatinine Ratio Panel	11.39	MOHL
Alkaline Phosphatase	1.57	MOHL
Alpha 1 Antitrypsin	20.57	MOHL
Alpha Aminoadipate/Creatinine	7.66	MOHL
Alpha Aminoadipic Semialdehyde (AASA) urine	257.95	MOHL
Alpha Glucosidase; Bld Spot	51.30	MOHL
Alpha-1 fetoprotein, centaur	24.75	MOHL
Amino Acids Panel; Blood	78.30	MOHL
Amino Acids Panel; CSF	324.09	MOHL
Amino Acids Panel; Urine	54.19	MOHL
Ammonia	7.40	MOHL
Amylase (all specimen types including CSF)	10.03	MOHL
APTT (activated partial thromboplastin time)	6.56	MOHL
Aspartate Aminotransferase	1.73	MOHL
Autoimmune/antibody testing –Other	58.80	Average
Bacterial autoantibody serum test (e.g. Treponema pallidum)	15.92	MOHL
Beta galactocerebrosidase	51.30	MOHL
Beta Galactosidase; WBC	51.30	MOHL
Beta Hydroxybutyrate	58.80	Average
Beta-Glucuronidase (MPS VII)	264.56	MOHL
Bile Acids, Total	32.31	MOHL
Bilirubin	1.56	MOHL
Biotinidase	264.56	MOHL
Blood urea nitrogen	7.41	MOHL
Blood, Culture (e.g. venous catheter)	11.75	MOHL
Bordetella species Investigation (whooping cough)	134.44	FindLabTest.com
Borrelia burgdorferi Ab (immunoblot - Lyme)	177.93	FindLabTest.com
C Peptide	47.35	MOHL
C Reactive Protein	10.29	MOHL
Calcium (all specimen types including CSF)	2.84	MOHL
Calcium Ionized (incl Panel)	14.18	MOHL
Carbamazepine	15.30	MOHL
Carbapenemase Producing Organisms; Culture	58.80	Average
Cardiolipin Ab IgG & IgM Panel	48.87	MOHL
Carnitine; Total	41.22	MOHL

© 2024 Weymann D et al. *JAMA Network Open.*

Item	Cost	Cost Source
Catecholamines/Creatinine	52.90	MOHL
CBC & Differential	10.94	MOHL
Cell Count; CSF	28.73	MOHL
Cerebroside Sulfatase AKA Aryl Sulfatase - Enzyme activity (leucocytes)	51.30	MOHL
Ceruloplasmin	10.13	MOHL
Chemistry Panel ((Na, K, Cl, Co2, Anion, Gluc rand, urea, Creatin, gFR))	25.29	MOHL
Chemistry Panel 2 ((protein, albumin, ALT, AST, Alk Phos, GGT, Bil))	12.19	MOHL
Chlamydia trachomatis+Neisseria gonorrhoeae DNA; Urine; PCR/NAAT	16.77	MOHL
Chloride (all specimen types including CSF)	4.82	MOHL
Chloride, sweat	77.38	MOHL
Cholesterol	6.86	MOHL
Choriogonadotropin Beta Subunit; Urine	15.48	MOHL
Clostridium difficile (C Difficile) Toxin; Stool	32.73	MOHL
Complement C3 or C4	50.08	FindLabTest.com
Copper	49.51	MOHL
CoQ measurement, muscle biopsy (e.g fibroblasts)	310.86	MOHL
Cortisol Morning	13.26	MOHL
Cortisol Random	77.13	MOHL
Creatine Kinase	1.88	MOHL
Creatine metabolites	66.55	FindLabTest.com
Creatinine	4.13	MOHL
Creatinine & Glomerular Filtration Rate Predicted Panel	4.13	MOHL
CSF: plasma glucose ratio	30.31	FindLabTest.com
Culture; CSF	15.28	MOHL
Cyclic Citrullinated Peptide Ab	29.44	MOHL
Cytomegalovirus Ab IgG, and DNA, PCR/NAAT	30.18	MOHL
DAT (Direct AntiGlobulin Testing - transfusion)	24.51	MOHL
Deoxypyridinoline	58.80	average
DHEA – dehydroepiandrosterone	18.52	MOHL
Differential; CSF	11.68	MOHL
DNA Double Strand Ab (autoantibody test0	55.36	FindLabTest.com
Drugs Identified; Urine; Screen	6.94	MOHL
Electrolyte, Urea & Creatinine Panel	18.90	MOHL
Electrolytes Panel	12.17	MOHL
Epstein Barr Virus Capsid Ab or DNA;PCR/NAAT	54.70	FindLabTest.com
Erythrocyte Sedimentation Rate	10.59	MOHL
Estradiol	22.40	MOHL
Extractable Nuclear Ab Panel	24.95	MOHL
Eye; Culture	15.28	MOHL
Factor VIII Activated Activity	51.13	MOHL
Factor XIII Clot Dissolution at 24h	51.50	MOHL
Fatty Acids Very Long Chain Pattern	91.55	MOHL
Fatty Aldehyde Dehydrogenase	22.76	MOHL

© 2024 Weymann D et al. *JAMA Network Open.*

Item	Cost	Cost Source
Ferritin	10.10	MOHL
Fibrin D Dimer FEU	23.24	MOHL
Fibrinogen	28.64	MOHL
Folate	38.22	FindLabTest.com
Follicle Stimulating Hormone FSH	13.11	MOHL
GAA guanidinoacetate – urine	58.80	Average
Gal-1-P-Uridyltransferase	70.73	MOHL
Galactosylceramidase; WBC	58.80	Average
Gamma Glutamyl Transferase (GGT)	1.66	MOHL
Gas Panel	22.01	MOHL
Gentamicin trough or peak	26.65	MOHL
Glucocerebrosidase - Enzyme activity (leucocytes)	51.30	MOHL
Glucose Fasting	26.73	MOHL
Glucose Random or non-Fasting (all specimen types)	6.40	MOHL
Glutamine	271.51	FindLabTest.com
Glycine	271.51	FindLabTest.com
Helicobacter pylori Ab IgG	36.02	MOHL
Hemoglobin Panel – electrophoresis	71.33	MOHL
Heparan-N-sulfatase (MPS IIIa)	264.56	MOHL
Hepatitis A Virus Ab	18.39	MOHL
Hepatitis B Virus	11.96	MOHL
Hepatitis C Virus Ab	94.90	FindLabTest.com
Herpes Simplex Virus 1+ 2 DNA; PCR/NAAT	51.57	MOHL
Herpes Virus 6 DNA; PCR/NAAT	79.08	FindLabTest.com
Heterophile Ab (Mononucleosis)	17.07	MOHL
hexosaminidase; total	67.25	MOHL
HIV 1+2 Ab+p24 Ag; Screen	51.57	MOHL
HLA-B27 (human leukocyte antigen) Related Ag	241.76	MOHL
Homocysteine; blood (total)	22.94	MOHL
Iduronate-2-sulfatase (MPS II)	264.56	MOHL
Iduronidase (MPS I)	51.17	MOHL
IgA, IgE, IgG, IgM (Immunoglobulin)	22.86	MOHL
INR and/or PT (Prothrombin Time) Panel	12.68	MOHL
Insulin	17.75	MOHL
Iron & Iron Binding Capacity Panel	7.55	MOHL
Iron Transferrin Saturation Panel	15.10	MOHL
Lactate	25.49	MOHL
Lactate Dehydrogenase	1.57	MOHL
lactate:pyruvate ratio; CSF	43.44	MOHL
lactate:pyruvate ratio; serum	210.88	FindLabTest.com
Lactate; CSF	1.06	MOHL
Lead; Bld	130.48	MOHL
Leukocytes	241.76	MOHL
Lipase	6.61	MOHL
Lipid Panel	52.30	MOHL
Luteinizing Hormone (LH)	12.39	MOHL
Magnesium	14.16	MOHL

© 2024 Weymann D et al. *JAMA Network Open.*

Item	Cost	Cost Source
Manganese; blood	156.84	FindLabTest.com
Mercury	52.34	MOHL
Microscopic Examination; Gram Stain	5.65	MOHL
mono-amine metabolites; urine	50.38	MOHL
MRSA, culture	51.57	MOHL
Mucopolysaccharides (or glycosaminoglycans)	59.46	MOHL
Muscle Specific Receptor Tyrosine Kinase Ab	245.23	MOHL
Myoglobin; Urine	117.30	FindLabTest.com
N-Acetyl-Glucosamine-6-sulfatase (MPS IIId)	264.56	MOHL
N-Acetyl-Glucosaminidase (MPS IIIb)	264.56	MOHL
Neopterin & Tetrahydrobiopterin; CSF	58.80	Average
Neurotransmitters, CSF	264.56	MOHL
Neutrophil Cytoplasmic Ab (incl Panel)	114.09	MOHL
Newborn screening panel - collection and result	182.20	MOHL
Nuclear Ab	25.07	MOHL
Oligoclonal Bands; CSF	31.16	MOHL
Oligosaccharides Pattern; Urine	58.80	Average
Organic Acids Panel; Urine	105.25	MOHL
Osmolality; Urine	49.26	MOHL
Ova & Parasites	43.87	MOHL
Oxysterols	58.80	Average
pH; Blood	58.80	Average
Phenobarbital	81.72	FindLabTest.com
Phenylalanine	20.53	MOHL
Phosphatase	1.57	MOHL
Phosphate	2.60	MOHL
Phosphatidylserine Ab IgG & IgM Panel	288.65	FindLabTest.com
phytanic acid	91.55	MOHL
Pipecolic Acid, urine	271.51	FindLabTest.com
Porphobilinogens; urine	9.69	MOHL
Potassium (all specimen types including CSF)	2.82	MOHL
Prealbumin	20.13	MOHL
Prolactin	13.47	MOHL
Proline	58.80	Average
Protein	2.63	MOHL
Protein Electrophoresis Panel (all specimen types including CSF)	30.51	MOHL
Protein; CSF	7.13	MOHL
PTH Intact	17.49	MOHL
Purines & Pyrimidines; Urine	63.24	MOHL
Pyruvate; Bld	17.95	MOHL
RAST- radioallergosorbent test	58.80	Average
Reducing substances; stool *	58.80	Average
Respiratory Chain Analysis	274.15	MOHL
Respiratory Panel, PCR/NAAT	58.80	Average
Reticulocytes	11.52	MOHL
Rheumatoid Factor	8.40	MOHL
Selenium	49.70	MOHL

© 2024 Weymann D et al. *JAMA Network Open.*

Item	Cost	Cost Source
Sjogrens Syndrome-A and B Ab	38.58	MOHL
Smith Ab and/or Ribonucleoprotein Ab	38.58	MOHL
Sodium (all specimen types including CSF)	2.55	MOHL
Sputum; Respiratory Culture	15.28	MOHL
Stool, culture	16.87	MOHL
T3 Free Request (Triiodothyronine)	9.34	MOHL
T4 Free (Thyroxine)	12.10	MOHL
T8	58.80	Average
Tetrahydrofolate; CSF	58.80	Average
Throat; Culture	18.18	MOHL
Thrombin time	8.31	MOHL
Thyroid Stimulating Hormone TSH	9.90	MOHL
Thyroperoxidase Ab	20.22	MOHL
Tissue Transglutaminase Ab - Panel or otherwise	13.92	MOHL
Toxoplasmosis Gondii Ab IgG or IgM	99.00	FindLabTest.com
Transferrin Isoelectric Focus	90.35	MOHL
Transfusion medicine report	58.80	Average
Triacylglycerol Lipase	51.17	MOHL
Triglycerides (fasting and non-fasting)	16.07	MOHL
Troponin I Cardiac	15.48	MOHL
Urate or uric acid	2.36	MOHL
Urea	2.59	MOHL
Urinalysis	10.27	MOHL
Urine; Culture	15.28	MOHL
Valproate/Valproic Acid	16.53	MOHL
Vancomycin Peak and/or Trough	15.55	MOHL
Virus Isolation (e.g. influenza, human pacherovirus, varicella zoster, rubella)	51.57	MOHL
Vitamin B12	14.36	MOHL
Vitamin E	53.86	MOHL
von Willebrand Factor (vWF) antigen or activity	107.50	MOHL
VRE culture	15.28	MOHL
Zinc	102.29	MOHL
Other Test	58.80	Average of all labs
**GENETIC TESTS AND PANELS**		
FISH (patient)	192.68	MSP
FISH parental testing (one parent)	466.46	MSP
FISH parental testing (two parents)	932.92	MSP
Karyotype	301.44	Dragojlovic et al. 2020[14]
Fragile X	624.06	LifeLabs
mtDNA Mutation/Deletion panel	871.19	Pacific Fertility
Chromosomal Breakage	181.81	MOHL
Spinocerebellar Ataxia	1002.28	BCCH
Spinomuscular Atrophy	255.00	Dragojlovic et al. 2020[14]
Myotonic Dystrophy Panel	1300.00	BCCH
ATP7A and ATP7B	2551.01	BCCH

© 2024 Weymann D et al. *JAMA Network Open.*

Item	Cost	Cost Source
SLC6A8	833.38	BCCH
Panel Periodic Fever Syndrome	890.00	BCCH
Other SGT (average)	960.13	Dragojlovic et al. 2020[14]
Whole Mitochrondrial DNA genome sequencing (mtDNA)	890.00	BCCH
Chromosomal Microarray	842.00	Jegathisawaran et al. 2019[15]
ACADM full gene sequencing	866.47	BCCH
Average Gene Panel	2619.91	Dragojlovic et al. 2020[14]
Muscular Dystrophy or Leukodystrophy Panel	2562.03	BCCH
Epilepsy Panel	2120.56	BCCH
Angelman Syndrome	5738.58	Commercial
Alagille Syndrome	2246.06	Dragojlovic et al. 2020[14]
Methylmalonic Acidemia Panel	1928.96	BCCH

MOHL: Ministry of Health Schedule of Fees for Laboratory Services; MSP: Ministry of Health Medical Services Commission Payment Schedule

© 2024 Weymann D et al. *JAMA Network Open.*

English Costing

100KGP individuals and hospital data

Within the 100KGP, de-identified information on each participant includes genome sequence data, phenotype and clinical data, administrative secondary care records (HES), and mortality data (Office of National Statistics (ONS)). The HES data can be linked to costs, as set out below, then each patient's activities in hospital and related costs are used in our main analyses. Available data in England spanned up to 11 years prior to GWS and up to 3 years after GWS.

NHS Hospital Episode Statistics (HES) data

NHS service providers collect and submit patient-level secondary care activity data to NHS Digital, which is collated and known collectively as Hospital Episode Statistics (HES), wherein each unit of activity is referred to as an episode. HES offer between 12 to 22 years of secondary care history across different data sets. Data on admitted patient care (APC), outpatient appointments (OP), and emergency attendances (A&E) are available from 1997, 2003 and 2007, respectively, until the end of 2019. HES provides information on the age of individuals, the date of the episode, the length of stay, the treatment speciality under which healthcare was provided, inter alia. Each episode in outpatient care and admitted patient care is defined by ICD-10 codes (International Statistical Classification of Diseases and Related Health Problems) and OPCS-4 codes (Classification of Interventions and Procedures).

NHS Reference Costs data

NHS Improvement (the administrative arm of the NHS performing a range of functions) regularly collects information from service providers who quantify their true costs. This information is published annually (the National Schedule of Reference Costs). This reference cost collection provides information on Healthcare Resource Group (HRG)-based unit costs that represent the unit costs to the NHS for providing defined services in a given financial year. These reference costs account for the direct costs of producing a healthcare service, indirect costs, and overheads.

Attaching costs to HES data

Reference costs were matched to HES data on an episode-by-episode basis. HES data were cleaned, then costs were allocated to each episode using specialised "Grouper" software version HRG4+ 2017/18. The software uses information on the episode to assign a HRG code. This includes condition and diagnostic information, treatment information, provider information and individual-specific information. National average unit costs from the National Schedule of Reference Costs 2017/18 were then linked to each episode-specific HRG. The same year was

© 2024 Weymann D et al. *JAMA Network Open.*

applied to all costs to avoid cost variation being attributed to year-to-year differences in reference cost procedures.

Inverse probability of censoring weighting

In Canadian analyses, we estimated inverse probability weights for each one-year time interval using Kaplan-Meier product limit estimates of probability of censoring.[12] In the English analyses, one-year interval inverse probability weights were generated with logistic regression. Inverse probability weighting reduces estimation bias by recreating the sample population expected in the absence of censoring.

Regression model specifications

The following equation represents the regression model used for our pre-post analysis:

$$E(c_{it}) = \exp\left(\alpha_i + \theta trend_t + \delta post_t + \beta X_{it} + \gamma_t + \varepsilon_{it}\right)$$

Where c_{it} are costs for participant i in year t; α_i are individual random effects; θ estimates the trend in costs over time, $post_t$ is a dummy variable denoting the post-GWS period; δ is the coefficient of interest and estimates the level change in costs post GWS, X_{it} is a vector of baseline covariates and β is vector of corresponding coefficients; γ_t are calendar year fixed effects; and ε_{it} is a standard error term capturing unexplained variation in the dependent variable. In pre-post analysis, this equation was fit using mixed effects generalized linear models estimated by maximum likelihood, assuming a log link and Gamma distribution. An independent correlation structure was assumed for random effects. Pre-post estimates reflect the estimated marginal effects of the level change on predicted mean costs for the fixed portion of the model leaving values of all other covariates as observed. Associated standard errors and confidence intervals were estimated using the Delta method.

The following equation represents the regression model used for our difference-in-differences analysis:

$$c_{it} = \alpha + \delta(post_t \times diagnosed_i) + \beta X_{it} + \theta diagnosed_i + \gamma_t + \varepsilon_{it}$$

Where c_{it} are costs for participant i in year t; α is an intercept term, ($post_t$ x $diagnosed_i$) is the interaction between a dummy variable denoting the post period and a dummy variable denoting the diagnosed group (so is a binary indicator equal to 1 for a diagnosed patient after GWS and 0 otherwise), δ is the coefficient of interest and estimates the effect of diagnosis on costs, X_{it} is a vector of baseline covariates and β is vector of corresponding coefficients; θ_i capture group differences at baseline, γ_t are calendar year fixed effects; and ε_{it} is a standard error term capturing unexplained variation in the dependent variable. In difference-in-differences analysis, this equation was fit using linear models to ensure interpretability of the coefficient of interest.[13]

In England, the final pre-post and difference-in-differences model adjusted for: age (continuously specified using natural splines), length of diagnostic odyssey (linearly specified); and categorical covariates of gender, ethnicity, deprivation decile, and region. In Canada, final models were stratified according to GWS setting (only-in-research vs. publicly reimbursed). In a BC research setting, the final pre-post model adjusted for: outcome trends (linearly specified), number of comorbidities, age at GWS (continuously specified with squared term), and random effects. The difference-in-differences model adjusted: calendar year and group fixed effects, number of comorbidities, sex, age at GWS (continuously specified with squared term), sex, and year of diagnostic odyssey in which GWS was accessed. In a BC publicly reimbursed setting, the final pre-post model adjusted for: outcome trends, number of comorbidities, age at GWS (continuously specified with squared term), phenotype, sex, area of residence, and random effects. The difference-in-differences model adjusted for: calendar year and group fixed effects, number of comorbidities, sex, age at GWS (continuously specified with squared term), sex, phenotype, and area of residence.

© 2024 Weymann D et al. *JAMA Network Open.*

eFigure 1: Study design diagram

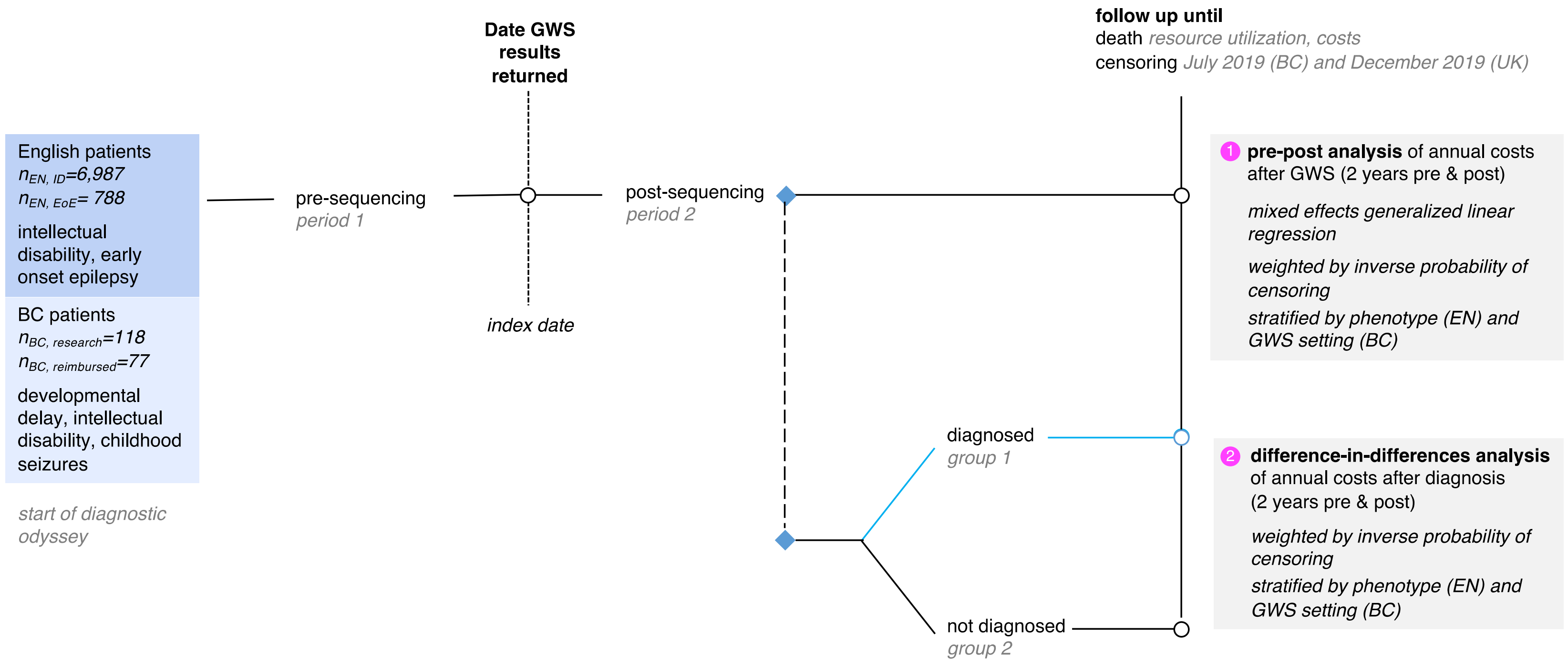

© 2024 Weymann D et al. *JAMA Network Open.*

eTable 1: Difference-in-difference regression estimates for 2 years pre and 2 years post in BC cohorts

	BC CAUSES Research Clinic (n=77) Diagnostic Yield: 55%			BC Publicly Reimbursed GWS (n=118) Diagnostic Yield: 40%		
Covariate	Coeff.	SE	p-value	Coeff.	SE	p-value
Intercept	1,691	530	0.005	2,618	522	<0.001
Post-GWS period flag	307	327	0.35	-2,015	323	<0.001
Diagnosed group flag	319	273	0.25	-542	294	0.07
Effect of diagnosis on costs	-736	391	0.06	74	462	0.87
Age at GWS	-356	92	<0.001	-409	119	0.001
Age at GWS, squared	18	5	<0.001	17	6	0.008
Sex, female	276	214	0.20	324	264	0.22
Area of residence						
Urban	-	-	-	Ref.		
Rural	-	-	-	-958	265	<0.001
GWS year of diagnostic odyssey	-62	42	0.14	-	-	-
Phenotype						
DD only	-	-	-	Ref.		
DD and seizures	-	-	-	437	235	0.07
Seizures only	-	-	-	1,199	479	0.01
No. of concomitant disorders	135	48	0.006	281	70	<0.001
Calendar year fixed effects	absorbed			absorbed		
Adjusted R^2	0.1428			0.2962		
Pr (F)	<0.001			<0.001		

© 2024 Weymann D et al. *JAMA Network Open.*

eTable 2: Difference-in-difference regression estimates for 2 years pre and 2 years post in English cohorts

	Early onset epilepsy (n=788, Diagnostic Yield 19%)			Intellectual disability (n=6,987, Diagnostic Yield 20%)		
	Coeff.	SE	p-value	Coeff.	SE	p-value
Intercept	7,976	1211	<0.001	2,652	249	<0.001
Post-GWS period flag	-605	540	0.27	-123	61	0.04
Diagnosed group flag	267	486	0.60	589	99	<0.001
Effect of diagnosis on costs	348	556	0.54	-86	82	0.30
Unknown yield	1,039	510	0.04	229	106	0.03
Neonate flag	4,707	629	<0.001	1,295	214	<0.001
Age of Onset	-1,426	478	0.003	-64	22	0.004
Age	-106	16	<0.001	-55	5	<0.001
Male	-348	359	0.34	-92	74	0.22
Black	867	1088	0.43	77	260	0.78
Asian	202	6176	0.76	291	127	0.02
Chinese	234	4885	0.97	-766	997	0.45
Mixed race	595	896	0.52	97	185	0.61
Other	-948	1471	0.53	-51	350	0.89
Not stated	-552	555	0.32	196	111	0.08
Region: Trent	-2,097	991	0.03	-303	164	0.07
Region: West Midlands	-1,356	962	0.16	143	174	0.42
Region: North West	-415	918	0.66	171	175	0.33
Region: Eastern	-1,664	915	0.07	-366	191	0.06
Region: London	-1,476	847	0.08	-156	175	0.38
Region: South East	-561	852	0.52	-26	160	0.88
Region: South west	-2,721	916	0.003	-590	188	0.002
IMD Least Deprived 10-20%	-356	794	0.67	155	169	0.37
IMD Least Deprived 20-30%	685	858	0.43	-56	179	0.77
IMD Least Deprived 30-40%	486	840	0.57	84	177	0.65
IMD Least Deprived 40-50%	989	769	0.20	231	175	0.19
IMD Most deprived 40-50%	-168	834	0.85	80	160	0.63
IMD Most deprived 30-40%	-146	805	0.87	122	165	0.47
IMD Most deprived 20-30%	149	819	0.87	149	167	0.38
IMD Most deprived 10-20%	525	823	0.53	145	169	0.40
IMD Most deprived 10%	-212	841	0.81	-97	159	0.55
2015	750	678	0.27	128	168	0.45
2016	-989	695	0.16	223	159	0.16
2017	-926	704	0.19	65	161	0.70
2018	-733	826	0.38	11	171	0.95
2019	-3,989	977	<0.001	-959	183	<0.001

© 2024 Weymann D et al. *JAMA Network Open.*

eTable 3: Logistic regression of probability of diagnosis from GWS, Canadian Cohorts

	BC CAUSES Research Clinic (n=77) Diagnostic Yield: 55%			BC Publicly Reimbursed GWS (n=118) Diagnostic Yield: 40%		
Covariate	OR	SE	p-value	OR	SE	p-value
GWS year of diagnostic odyssey	0.97	0.11	0.81	1.08	0.09	0.38
Age at GWS	1.07	0.10	0.49	0.92	0.05	0.13
Sex, female	1.51	0.80	0.44	2.06	0.83	0.07
Area of residence						
Urban	Ref.			Ref.		
Rural	0.93	0.67	0.92	0.89	0.87	0.91
Phenotype						
DD only	Ref.			Ref.		
DD and seizures	1.37	0.82	0.60	0.91	0.43	0.84
Seizures only	1.38	2.08	0.83	0.39	0.24	0.13
No. of concomitant disorders	1.38	0.20	0.02	0.96	0.11	0.74
Intercept	0.13	0.12	0.02	0.76	0.49	0.67
Pseudo R^2	0.09			0.054		
Log likelihood	-48.27			-74.16		
Pr (Chi2)	0.21			0.29		

OR: adjusted odds ratio; BCD: Biochemical Diseases; CAUSES: Clinical Assessment of the Utility of Sequencing and Evaluation as a Service; SE: Standard error; GWS: genome-wide sequencing, DD: developmental delay. Significance level: $p<0.05$

Interpretation: Few baseline differences across diagnosed and not diagnosed patients were statistically significant in either a research or publicly reimbursed Canadian setting. Research participants with more concomitant disorders were significantly more likely to be diagnosed from GWS (aOR: 1.38, p=0.02).

© 2024 Weymann D et al. *JAMA Network Open.*

eTable 4: Logistic regression of probability of diagnosis from GWS, United Kingdom Cohorts

	Early onset epilepsy (n=788, Diagnostic Yield 19%)			Intellectual disability (n=6,987, Diagnostic Yield 20%)		
	OR	SE	sig	OR	SE	sig
Age of Onset	1.06	0.28		0.88	0.04	***
Age	0.99	0.01		0.99	0.00	
Male	0.69	0.21		0.70	0.07	***
Black	1.07	0.62		0.92	0.26	
Asian	1.08	0.37		1.01	0.12	
Chinese	1.40E-05	535.41		5.53	0.78	*
Mixed race	0.76	0.58		0.88	0.18	
Other	3.86	0.75		1.57	0.30	
Not stated	0.62	0.35		1.02	0.10	
Region: Trent	0.73	0.62		1.17	0.16	
Region: West Midlands	0.58	0.61		1.22	0.17	
Region: North West	1.27	0.52		1.14	0.17	
Region: Eastern	1.97	0.53		1.23	0.18	
Region: London	1.04	0.51		1.26	0.17	
Region: South East	1.05	0.51		1.04	0.16	
Region: South west	1.52	0.53		1.21	0.18	
IMD Least Deprived 10-20%	0.58	0.47		0.81	0.15	
IMD Least Deprived 20-30%	0.60	0.50		0.80	0.16	
IMD Least Deprived 30-40%	0.70	0.46		0.61	0.16	**
IMD Least Deprived 40-50%	0.59	0.45		0.52	0.16	***
IMD Most deprived 40-50%	0.62	0.45		0.59	0.16	***
IMD Most deprived 30-40%	0.79	0.47		0.51	0.16	***
IMD Most deprived 20-30%	1.06	0.48		0.54	0.16	***
IMD Most deprived 10-20%	0.63	0.50		0.66	0.15	**
IMD Most deprived 10%	0.98	0.46		0.56	0.15	***

OR: adjusted odds ratio; SE: Standard error; IMD: indices of deprivation. Significance level: $p<0.05$

Interpretation: Probability of EOE diagnosis from GWS was unrelated to age, gender, ethnicity, region, socioeconomic status, or length of diagnostic odyssey. The probability of ID diagnosis from GWS was statistically significantly associated with male (reference: female, aOR: 0.70 ; $p<0.05$), Chinese ethnicity (reference: white ethnicity, aOR: 5.52; $p<0.05$), lower deciles of socioeconomic status (reference: upper decile of socioeconomic status, aOR: 0.61-0.56; $p<0.05$), and lower age of onset (aOR: 0.87; $p<0.05$).

© 2024 Weymann D et al. *JAMA Network Open.*

eFigure 2: Unadjusted annual total cost trajectory across cohorts

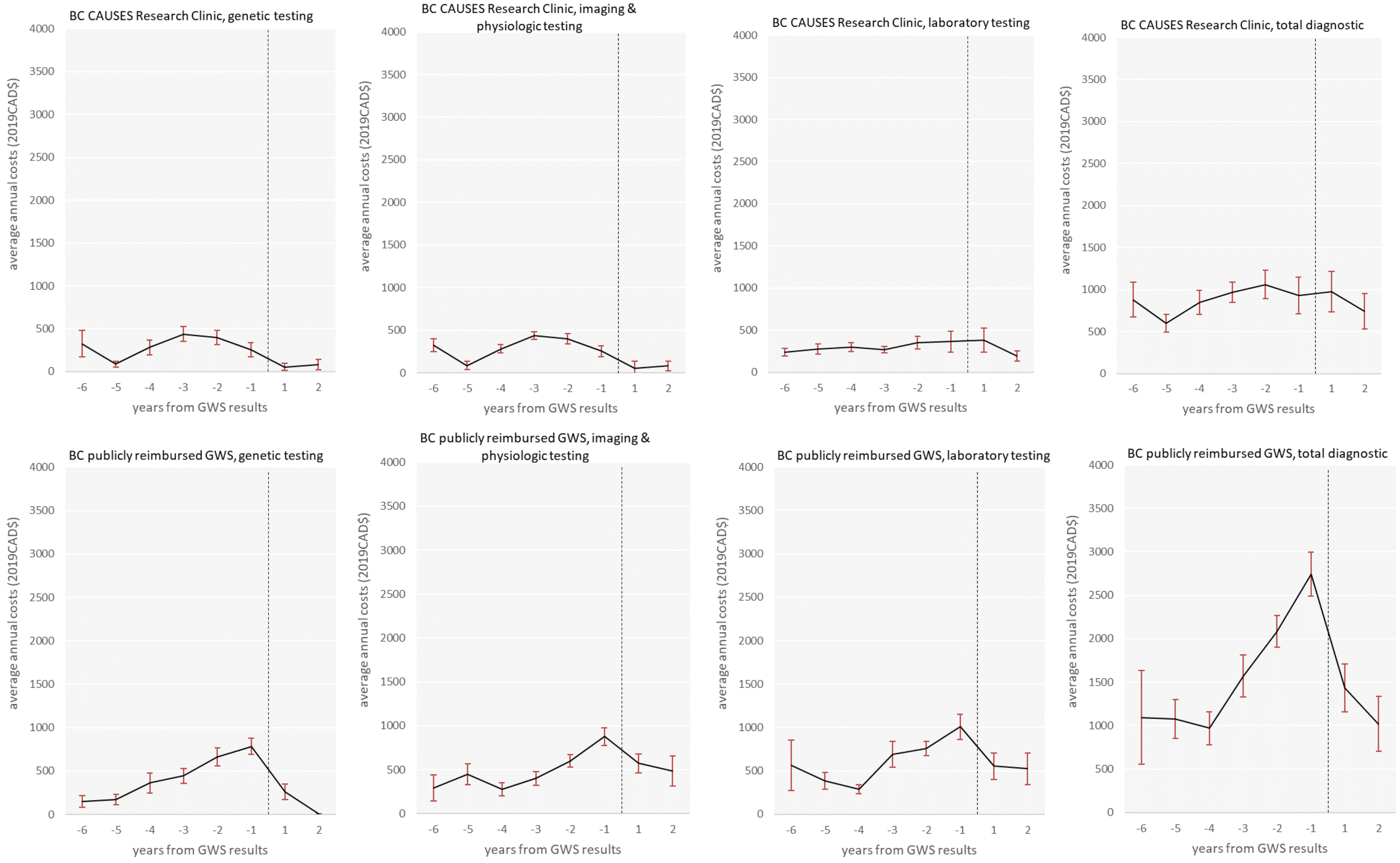

© 2024 Weymann D et al. *JAMA Network Open.*

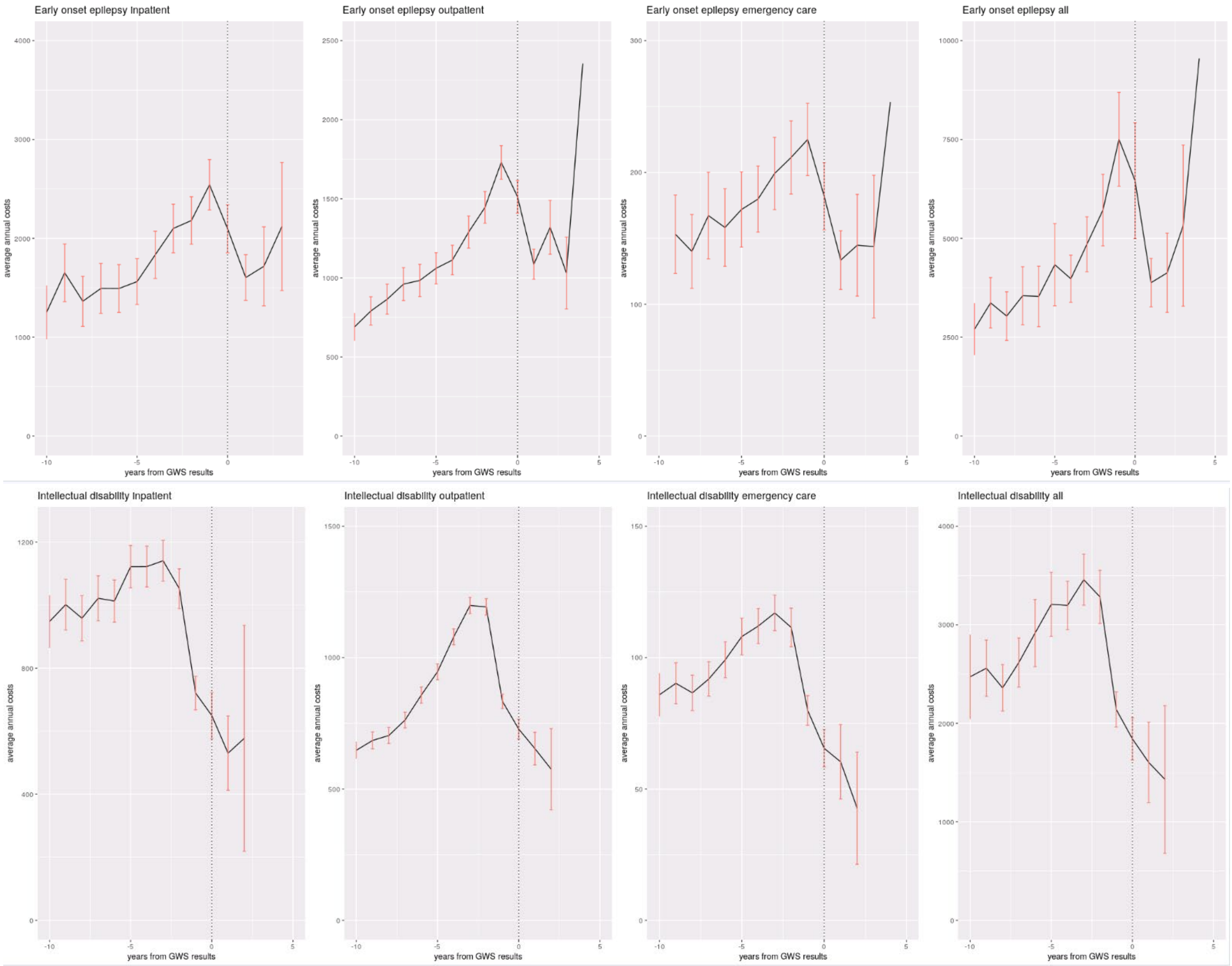

Unadjusted for covariates or censoring, mean annual costs reported in 2019 $CAD or £GBP for complete cases only.

© 2024 Weymann D et al. *JAMA Network Open.*

eTable 5: Unadjusted annual costs over 2 years pre and 2 years post period, Canada

Annual costs over 2 years pre and 2 years post period ($CAD)	BC Publicly Reimbursed GWS (n=118, Diagnostic Yield 40%)		BC CAUSES Research Clinic (n=77, Diagnostic Yield 55%)	
	Mean	SE	Estimate	SE
All diagnostic costs	2,087	135	961	109
Genetic testing costs	555	53	226	39
Imaging and physiological testing costs	689	884	320	36
Laboratory testing costs	785	73	345	59

Abbreviations: CAUSES: Clinical Assessment of the Utility of Sequencing and Evaluation as a Service; GWS: genome-wide sequencing; SE: standard error

Unadjusted for covariates or censoring, mean annual costs reported in 2019 $CAD or £GBP for complete cases only.

eTable 6: Unadjusted annual costs over 2 years pre and 2 years post period, England

Annual costs over 2 years pre and 2 years post period (£GBP)	English 100,000 Genomes Project, Early Onset Epilepsy (n=788, Diagnostic Yield 19%)		English 100,000 Genomes Project, Intellectual Disability (n=6,987, Diagnostic Yield 20%)	
	Mean	SE	Estimate	SE
All care costs	4,138	65	2,642	20
Inpatient costs	1,954	43	986	13
Outpatient costs	1,300	18	991	6
Emergency costs	182	5	101	2

Abbreviations: GBP: Great British pound; SE: standard error

Unadjusted for covariates or censoring, mean annual costs reported in 2019 $CAD or £GBP for complete cases only.

eFigure 3: Unadjusted annual total cost trajectory across cohorts, stratified according to diagnosis from GWS

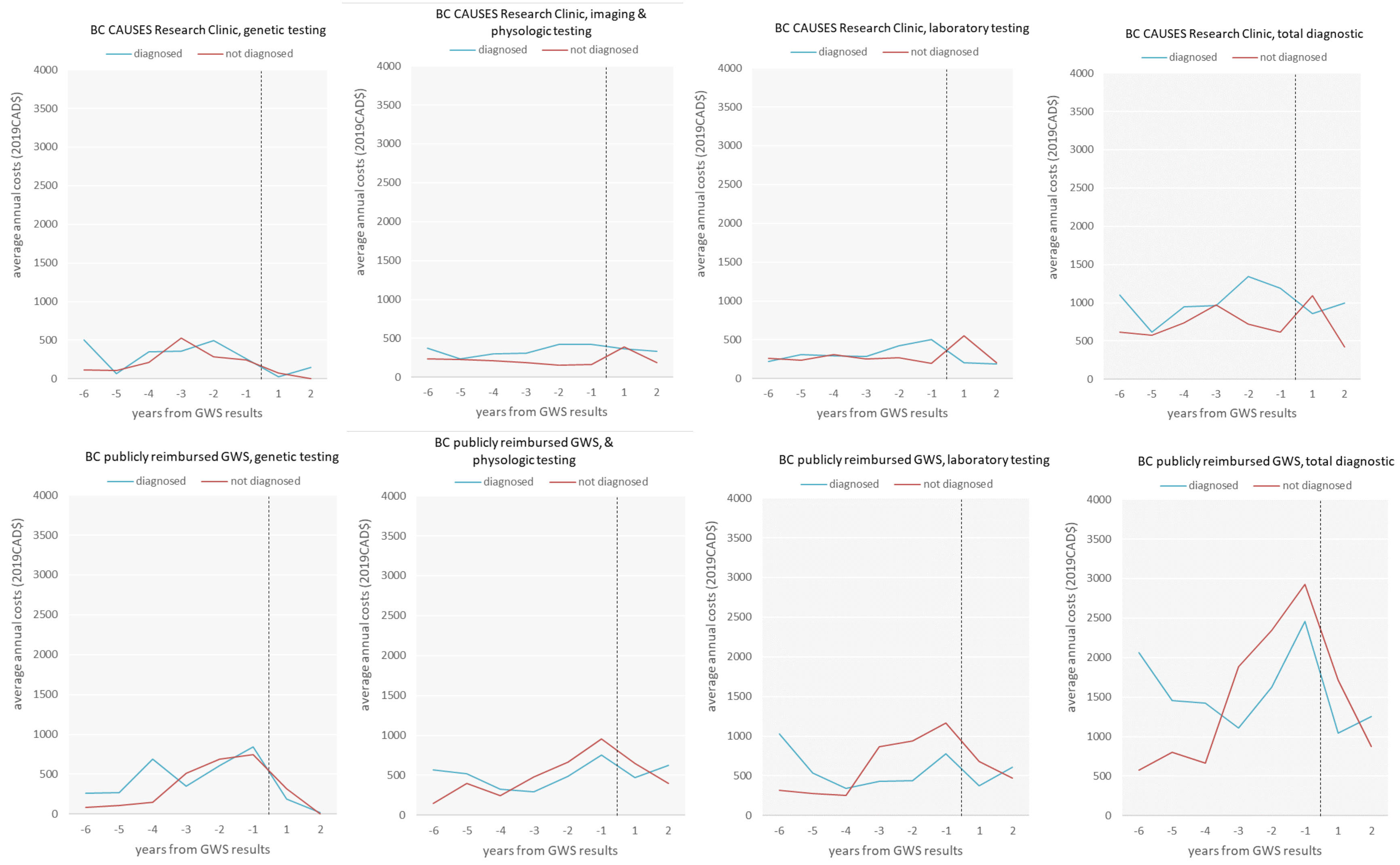

© 2024 Weymann D et al. *JAMA Network Open.*

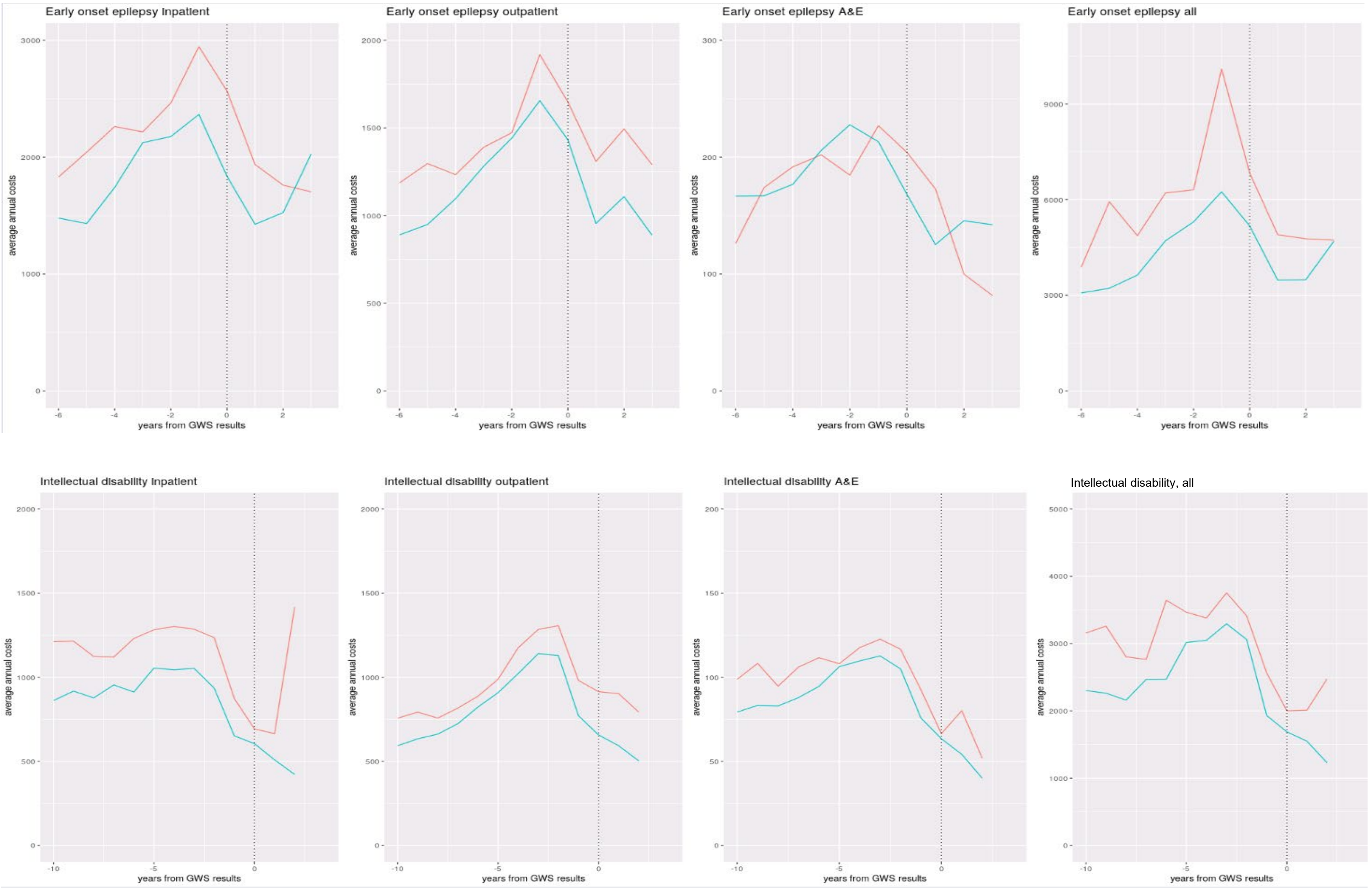

Footnote: Unadjusted for covariates or censoring, mean annual costs reported in 2019 $CAD or £GBP for complete cases only

© 2024 Weymann D et al. *JAMA Network Open.*

eTable 7: Sensitivity analysis considering 1 year pre and 1 year post-GWS

	English 100,000 Genomes Project EoE (n=788, Diagnostic Yield 19%)			English 100,000 Genomes Project ID (n=6,987, Diagnostic Yield 20%)			BC Publicly Reimbursed GWS (n=118, Diagnostic Yield 40%)			BC CAUSES Research Clinic (n=77, Diagnostic Yield 55%)		
Regression model	Estimate	SE	p-value	Estimate	SE	p-value	Estimate	SE	p-value	Estimate	SE	p-value
Pre-post change following GWS	£553	464	0.24	£140	59	0.02	-$2,248	390	<0.001	$60	197	0.76
Effect of diagnosis from GWS	£83	650	0.91	£106	105	0.32	-$274	575	0.64	-$920	427	0.03

Abbreviations: EoE: early onset epilepsy; ID: intellectual disability; GWS: genome-wide sequencing; Incr. incremental; SE: standard error. Significance level: p<0.05. Costs reported in 2019 $CAD or £GBP

eTable 8: Sensitivity analysis considering 6 years pre and 2 years post-GWS

	English 100,000 Genomes Project EOE (n=788, Diagnostic Yield 19%)			English 100,000 Genomes Project ID (n=6,987, Diagnostic Yield 20%)			BC Publicly Reimbursed GWS (n=118, Diagnostic Yield 40%)			BC CAUSES Research Clinic (n=77, Diagnostic Yield 55%)		
Regression model	Estimate	SE	p-value	Estimate	SE	p-value	Estimate	SE	p-value	Estimate	SE	p-value
Pre-post change following GWS	£447	498	0.37	£83	42	0.04	-$1,770	257	<0.001	$185	207	0.37
Effect of diagnosis from GWS	-£68	466	0.82	-£41	72	0.58	$74	462	0.87	-$736	391	0.06

Abbreviations: EoE: early onset epilepsy; ID: intellectual disability; GWS: genome-wide sequencing; Incr. incremental; SE: standard error. Significance level: p<0.05. Costs reported in 2019 $CAD or £GBP.

© 2024 Weymann D et al. *JAMA Network Open.*

References

1. 100 GPPI. 100,000 genomes pilot on rare-disease diagnosis in health care—Preliminary report. *New England Journal of Medicine.* 2021;385(20):1868-1880.
2. Eltze CM, Chong WK, Cox T, et al. A population-based study of newly diagnosed epilepsy in infants. *Epilepsia.* 2013;54(3):437-445.
3. Elliott AM, du Souich C, Adam S, et al. The Genomic Consultation Service: A clinical service designed to improve patient selection for genome-wide sequencing in British Columbia. *Molecular Genetics & Genomic Medicine.* 2018;6(4):592-600.
4. Elliott AM, Adam S, du Souich C, et al. Genome-wide sequencing and the clinical diagnosis of genetic disease: The CAUSES study. *Human Genetics and Genomics Advances.* 2022;3(3).
5. Authority MOHAPHS. Out of Province/Out of Country Laboratory and Genetic Testing Policy and Guidelines. http://www.bccss.org/bcaplm-site/Documents/Updates/Policy-guidelines-OOP-OOC-Lab-Genetic-Testing.pdf. Published 2021. Accessed 31/05/2022.
6. Canada S. T1 Family File, Final Estimates, 2016

Section 4 - Geography. https://www150.statcan.gc.ca/n1/pub/72-212-x/2018001/sect4-eng.htm. Published 2016. Accessed 22/09/2023.

7. Canada S. Variant: Population Centre and Rural Area 2016 by Province and Territory. https://www23.statcan.gc.ca/imdb/p3VD.pl?Function=getVDStruct&TVD=340985&CVD=314302&CPV=59&CST=01012016&CLV=2&MLV=4. Published 2016. Accessed 22/09/2023.
8. CADTH. *Guidance document for the costing of health care resources in the Canadian setting. 2nd edition.* Ottawa: CADTH;2016.
9. Ministry of Health. Medical Services Commission Payment Schedule. B.C. Ministry of Health. https://www2.gov.bc.ca/assets/gov/health/practitioner-pro/medical-services-plan/msc-payment-schedule-november-2019.pdf. Published 2019. Accessed 26/04/2022.
10. Schedule of Fees For the Laboratory Services Outpatient Payment Schedule. B.C. Ministry of Health. http://www.bccss.org/bcaplm-site/Documents/Programs/laboratory_services_schedule_of_fees.pdf. Published 2019. Accessed 26/04/2022.
11. Medical Services Plan MSP Fee-For-Service Payment Analysis: 2016/2017 - 2020/2021. B.C. Ministry of Health. https://www2.gov.bc.ca/assets/gov/health/practitioner-pro/medical-services-plan/msp_ffs_payment_analysis_20162017_to_20202021.pdf. Published 2021. Accessed 26/04/2022.
12. Kaplan EL, Meier P. Nonparametric estimation from incomplete observations. *Journal of the American statistical association.* 1958;53(282):457-481.
13. Puhani PA. The treatment effect, the cross difference, and the interaction term in nonlinear "difference-in-differences" models. *Economics Letters.* 2012;115(1):85-87.
14. Dragojlovic N, van Karnebeek CD, Ghani A, et al. The cost trajectory of the diagnostic care pathway for children with suspected genetic disorders. *Genetics in Medicine.* 2020;22(2):292-300.
15. Jegathisawaran J, Tsiplova K, Ungar WJ. A microcosting and cost-consequence analysis of genomic testing strategies (including trios) in autism spectrum disorder: an update. *The Hospital for Sick Children: Technology Assessment at SickKids.* 2019.

© 2024 Weymann D et al. *JAMA Network Open.*
