# Supplementary material for: Health Care Costs After Genome-Wide Sequencing for Children With Rare Diseases in England and Canada
Source: JAMA Netw Open. 2024 Jul 10;7(7):e2420842. doi: 10.1001/jamanetworkopen.2024.20842 (PMC11238031; doi:10.1001/jamanetworkopen.2024.20842)
Supplement: Supplement 2. — Data Sharing Statement [file jamanetwopen-e2420842-s002.pdf]

Data Sharing Statement

Weymann. Health Care Costs After Genome-Wide Sequencing for Children With Rare Diseases in England and Canada. JAMA Netw Open. Published July 10, 2024. doi:10.1001/jamanetworkopen.2024.20842

Data
Data available: No

Additional Information
Explanation for why data not available: Patient-level data used in this retrospective study are confidential and are not available in a public repository, in accordance with institutional policies. Data must be requested directly from institutional data stewards at Genomics England and the Provincial Health Services Authority.
